# Bug off or bug out: mapping flight secrets of *Triatoma garciabesi* (Hemiptera: Reduviidae) through climate, geography, and greenery

**DOI:** 10.3389/finsc.2025.1532298

**Published:** 2025-01-28

**Authors:** Thaiane Verly, Federico G. Fiad, Ana Laura Carbajal-de-la-Fuente, Sebastián Pita, Romina V. Piccinali, Patricia A. Lobbia, Paz Sánchez-Casaccia, Antonieta Rojas de Arias, María José Cavallo, Gisel V. Gigena, Claudia S. Rodríguez, María C. Vega-Gómez, Miriam Rolón, Julieta Nattero

**Affiliations:** ^1^ Centro Nacional de Diagnóstico e Investigación en Endemo-Epidemias (CeNDIE), Administración Nacional de Laboratorios e Institutos de Salud “Dr. Carlos Malbrán” (ANLIS), Buenos Aires, Argentina; ^2^ Consejo Nacional de Investigaciones Científicas y Técnicas (CONICET), Buenos Aires, Argentina; ^3^ Cátedra de Morfología Animal, Instituto de Investigaciones Biológicas y Tecnológicas (IIByT), Facultad de Ciencias Exactas Físicas y Naturales, Consejo Nacional de Investigaciones Científicas y Técnicas (CONICET)/Universidad Nacional de Córdoba, Córdoba, Argentina; ^4^ Sección Genética Evolutiva, Facultad de Ciencias, Universidad de la República, Montevideo, Uruguay; ^5^ Departamento de Ecología Genética y Evolución, Laboratorio de Eco-Epidemiología, Facultad de Ciencias Exactas y Naturales, Universidad de Buenos Aires, Ciudad Autónoma de Buenos Aires, Argentina; ^6^ Instituto de Ecología, Genética y Evolución (IEGEBA), CONICET-Universidad de Buenos Aires, Ciudad Autónoma de Buenos Aires, Argentina; ^7^ Unidad Operativa de Vectores y Ambiente (UnOVE), Centro Nacional de Diagnóstico e Investigación en Endemo-Epidemias (CeNDIE), Administración Nacional de Laboratorios e Institutos de Salud “Dr. Carlos Malbrán, Santa María de Punilla, Córdoba, Argentina; ^8^ Centro para el Desarrollo de la Investigación Científica (CEDIC), Asunción, Paraguay; ^9^ Centro Regional de Energía y Ambiente para el Desarrollo Sustentable (CREAS-CONICET)-Universidad Nacional de Catamarca (UNCA), San Fernando del Valle de Catamarca, Catamarca, Argentina; ^10^ Departamento de Biodiversidad y Biología Experimental. Facultad de Ciencias Exactas y Naturales, Universidad de Buenos Aires, Ciudad Autónoma de Buenos Aires, Argentina

**Keywords:** forewing, head, geometric morphometry, size and shape variation, climatic variables, geographic variables, vegetation cover

## Abstract

**Introduction:**

*Triatoma garciabesi* is a vector of *Trypanosoma cruzi*, the causative agent of Chagas disease, and is found across northwest and central Argentina, southern Bolivia, and western Paraguay. It frequently invades rural houses during the warm seasons and is common in peridomestic and wild environments. Recently, the existence of two lineages has been demonstrated based on variation in cytochrome *c* oxidase I gene (*coI*). These lineages occur across the species distribution range and coincide with different ecological regions. Here, we aim to examine how phenotypic variation in flight-related traits is structured, determine the association between these traits and geographic distance, and identify the climatic, geographic, and/or vegetation cover variables that best explain the morphometric variation in flight-related traits.

**Methods:**

A total of 198 males of *T. garciabesi* from 24 populations in Argentina and Paraguay were included in this study, covering almost the entire *T. garciabesi* distribution range. Size and shape components of the forewing, membranous, and stiff portions of the forewing and head were measured using a landmark-based methodology.

**Results:**

Our study documents that the size component of the membranous and stiff portions showed significant variation across the species range. Although forewing and head shape did not show significant differences in Procrustes distances across all pairs of populations, the membranous and stiff portions did. There is a strong and consistent association between shape and geographic distances at all levels of comparison (species and lineage ranges). The size and shape components and the geographic, climatic, and/or vegetation indexes explained covariation in all flight-related traits.

**Discussion:**

*T. garciabesi* appears to be a species sensitive to vegetation cover and landscape features. This study provides evidence for this by showing clear variation in flight-related traits across the species and lineage distribution range, as well as indications of isolation by distance and variation in flight-related traits according to climate, geography, and vegetation cover.

## Background

The triatomines (Hemiptera: Reduviidae: Triatominae), commonly known as kissing bugs, are capable of transmitting *Trypanosoma cruzi* (Chagas, 1909), the etiological agent of Chagas disease, primarily through contact with the feces of infected blood-sucking insects. It is estimated that 6 to 7 million people worldwide are infected with *T. cruzi*, 75 million people are considered at risk of infection, and the disease causes approximately 12,000 deaths annually (1). Hence, studying the ecology, distribution, and behavior of this subfamily is essential for enhancing control measures and mitigating the impact of Chagas disease. The subfamily Triatominae is composed of approximately 160 known species, grouped into five tribes and 18 genera (2). Among these, the genus *Triatoma* is notable for its morphological diversity and for containing the largest number of species (~80 species) (3, 4). One of the species of interest is *Triatoma garciabesi* Carcavallo, Cichero, Martínez, Prosen, Ronderos, 1967, found across northwest and central Argentina, southern Bolivia, and western Paraguay where it frequently invades rural houses during the summer season (5–8). Moreover, Canale et al. (9) reported a higher abundance of *T. garciabesi* in peridomestic ecotopes associated with the rugged bark of *Prosopis* sp., following the drastic reduction in the presence of *Triatoma infestans* (Klug, 1834). In sylvatic ecotopes, this species is commonly found in birds and rodent nests, hollow trees, loose tree barks, and occasionally epiphytic and terrestrial bromeliads (10).

In triatomines, head and forewings play a crucial role as fundamental structures during flight dispersal; narrow heads, with greater development of compound eyes, have been proposed as a characteristic that enhances flight performance (11–13). Heteroptera forewings (hemelytra) consist of two distinct zones: a stiff proximal zone and a more flexible membranous apex. The anterior sclerotized zone provides structural support and regulates forewing deformation, while the distal membranous zone is more easily deformed by aerodynamic and inertial forces (14). An increase in the size of this zone may suggest enhanced flight performance (14). In insects, small changes in wing shape may affect aerodynamic performance and, consequently, flight capacity (15). Those with elongated wings are suited for fast, straight long-distance flight, while shorter, broader wings are optimal for slow, agile short-distance flight (16). Landscape characteristics may drive the adaptations of flight-related traits to improve dispersal efficiency (17). As a result, vegetation covers and the climatic factors that influence it may act as environmental selective pressure that shapes phenotypic adaptations associated with flight traits (18, 19).

Specific climatic conditions can influence the occurrence of triatomines (20). Several studies have shown a positive association between temperature- and precipitation-related variables and the distribution of various triatomine species, including *Rhodnius nasutus* Stål, 1859, *Rhodnius neglectus* Lent, 1954 (21), *Triatoma recurva* (Stål, 1868) (22), *Triatoma sanguisuga* (LeConte, 1855) (23), *Panstrongylus geniculatus* (Latreille, 1811), *Rhodnius pallescens* Barber, 1932, and *Rhodnius prolixus* Stål, 1859 (24). Gigena et al. (13) found that geographic and climatic factors are associated with forewing and head size and shape in *Triatoma guasayana* Wygodzinsky and Abalos, 1949. A recent study revealed significant correlations between temperature, precipitation, and latitude with the forewing size and shape of *T. infestans*, indicating that forewing morphology is also influenced by geographic distribution (25).

Recently, Verly et al. (26) demonstrate the existence of two distinct lineages based on variation in cytochrome *c* oxidase I gene (*coI*). The *coI* gen has been widely used to improve understanding of species diversity and delimitation in phylogenetic and population genetics studies in insect species (27). Moreover, this mitochondrial marker has been greatly utilized in triatomines (28 and references therein). Lineages reported by Verly et al. (26), referred to as Eastern and Western, occur across the species distribution range, coincide with different ecological regions, and exhibit a mean genetic pairwise distance of 3.2% (kimura 2p adjusted) between them. However, this *coI* genetic distance is not sufficient to consider them as distinct species (26). Morphometry variation of flight-related traits such as the head, pronotum, and forewing aligns with lineage differentiation found with *coI* (26). In addition, another study showed that environmental changes impose selective pressures on *T. garciabesi* populations, resulting in a larger body, head, and forewing size in individuals in more anthropized landscapes compared to less anthropized areas, which may affect flight dispersal (19).

In this study, our aims were to (i) examine how *T. garciabesi* phenotypic variation in flight-related traits is structured, (ii) determine the association between these traits and geographic distance, and (iii) identify the climatic, geographic, and/or vegetation cover variables that best explain the morphometric variation in flight-related traits.

## Methods

### Insects and collection sites

**Figure 1:**
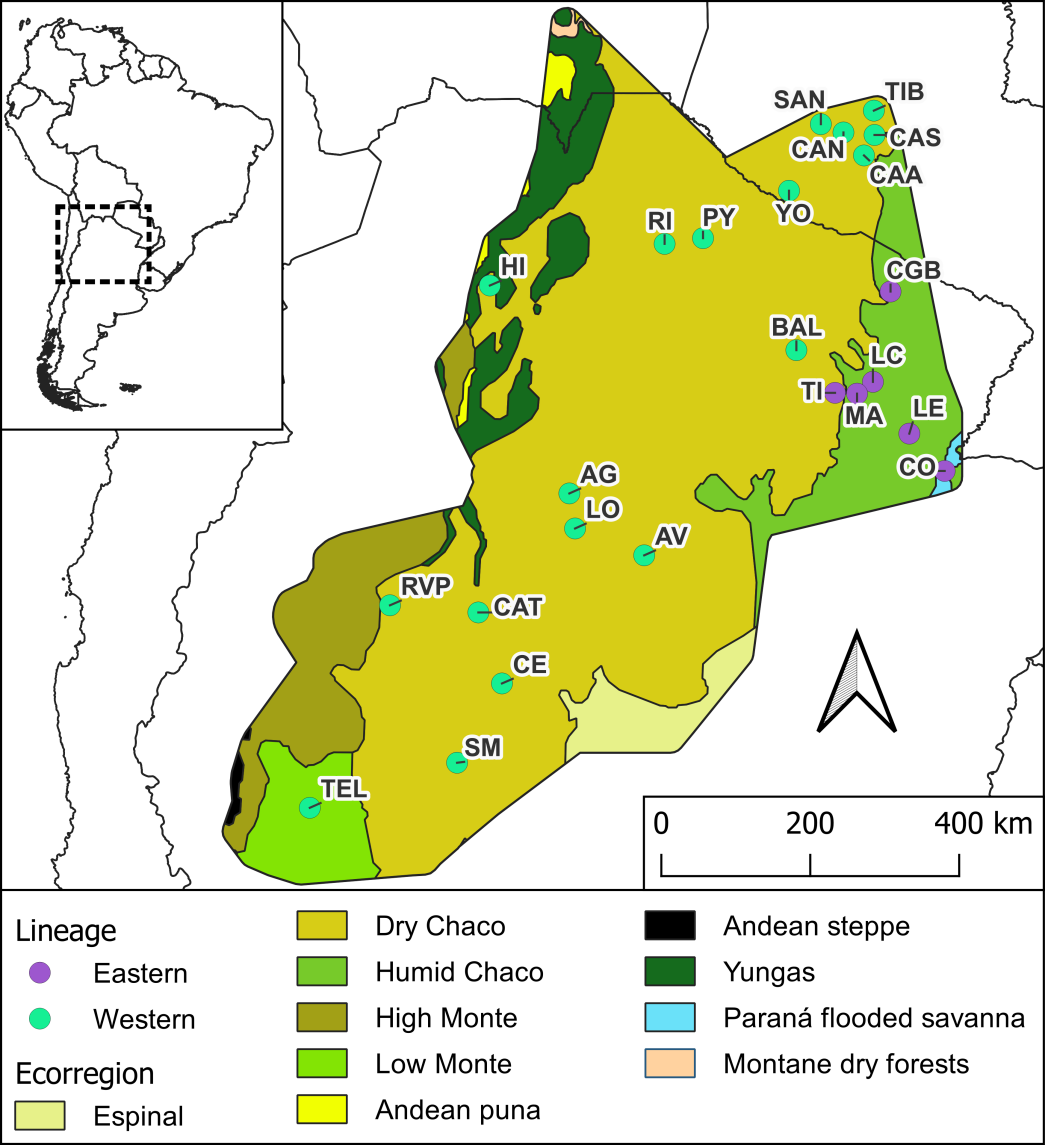
Distribution area of *Triatoma garciabesi.* Filled circles indicate the 24 locations of the studied populations, with purple circles corresponding to the populations of the Eastern lineage and green circles corresponding to populations assigned to the Western lineage. Populations codes as in **Table 1**. Ecoregions are according to Dinerstein et al. (29) (https://ecoregions.appspot.com/).

Males of *T. garciabesi* from 24 populations across Argentina and Paraguay were included in this study, covering nearly the entire distribution range (**Table 1**). Owing to morphological similarities between *T. garciabesi*, *Triatoma sordida* (Stål, 1859), and *Triatoma rosai* Alevi, Oliveira, Garcia, Cristal, Delgado, Bittinelli, Reis, Ravazi, Oliveira, Galvăo, Azeredo-Oliveira, Madeira, 2020, male identification was confirmed using molecular techniques for one or two individuals from each population. For this, *coI* gene was used, and total DNA was extracted from the legs fixed in 70% ethanol using a standard phenol–chloroform technique procedure. An approximately 624-bp coI fragment was amplified by PCR and the products were sent to Macrogen Inc. (Seoul, Korea) for DNA purification and subsequent sequencing. Both sequence strands were aligned and manually curated by chromatogram evaluation using Chromas (https://technelysium.com.au/wp/chromas/). A phylogenetic tree using the maximum likelihood (ML) method was done and the best-fitting substitution model under the Bayesian Information Criterion (BIC) was used (for more details see 26). The origin (field or colony reared in the laboratory) and the collection site (peridomestic or sylvatic) of the populations included in this study were described elsewhere (26). Only males were included in this study due to the sexual dimorphism observed in morphometric traits for this species (19). *T. garciabesi* distribution encompasses five ecoregions where the Eastern and Western lineages are found (26). The Eastern lineage is restricted to a portion of the center and eastern part of the Argentine provinces of Chaco and Formosa, predominantly within the Humid Chaco ecoregion, with one population near the boundary between the Humid and Dry Chaco and another in the Paraná Flooded Savanna (**Figure 1**). The Western lineage is present throughout the remainder of the species distribution range, with most of the populations being located in the Dry Chaco ecoregion with a few populations in the High and Low Monte ecoregions. These ecoregions exhibited a marked precipitation variation and significant differences in phytogeographical characteristics (30).

**Table 1:** Group defined with *coI*, geographical location, origin and individual number of each collection site for *Triatoma garciabesi* studied populations.

Lineages	Population name	Population code	Province/State	Country	N° of individuals
Eastern	3 Isletas	TI	Chaco	Argentina	8
Eastern	Corrientes	CO	Corrientes	Argentina	5
Eastern	Crucero Gral Belgrano	CGB	Formosa	Argentina	21
Eastern	La Esperanza	LE	Chaco	Argentina	8
Eastern	Lote 4	LC	Chaco	Argentina	5
Eastern	Maipú	MA	Chaco	Argentina	6
Western	Aguirre	AG	Stgo del Estero	Argentina	9
Western	Avellaneda	AV	Stgo del Estero	Argentina	10
Western	Balbuena	BAL	Chaco	Argentina	9
Western	Balde de Punta	CAT	Catamarca	Argentina	17
Western	Caacupé	CAA	Boquerón	Paraguay	5
Western	Canausa	CAN	Boquerón	Paraguay	5
Western	Casuarina	CAS	Boquerón	Paraguay	6
Western	Cruz del Eje	CE	Córdoba	Argentina	15
Western	Gral San Martín	SM	La Rioja	Argentina	9
Western	Hickman	HI	Salta	Argentina	5
Western	Loreto	LO	Stgo del Estero	Argentina	5
Western	Pozo Yacaré	PY	Formosa	Argentina	5
Western	Reserva Telteca	TEL	Mendoza	Argentina	6
Western	Rivadavia	RI	Salta	Argentina	8
Western	Rosario Vera Peñaloza	RVP	La Rioja	Argentina	10
Western	Sandhort	SAN	Boquerón	Paraguay	7
Western	Tiberia	TIB	Boquerón	Paraguay	7
Western	Yotoisha	YO	Boquerón	Paraguay	7

### Data collection on flight-related traits

**Figure 2:**
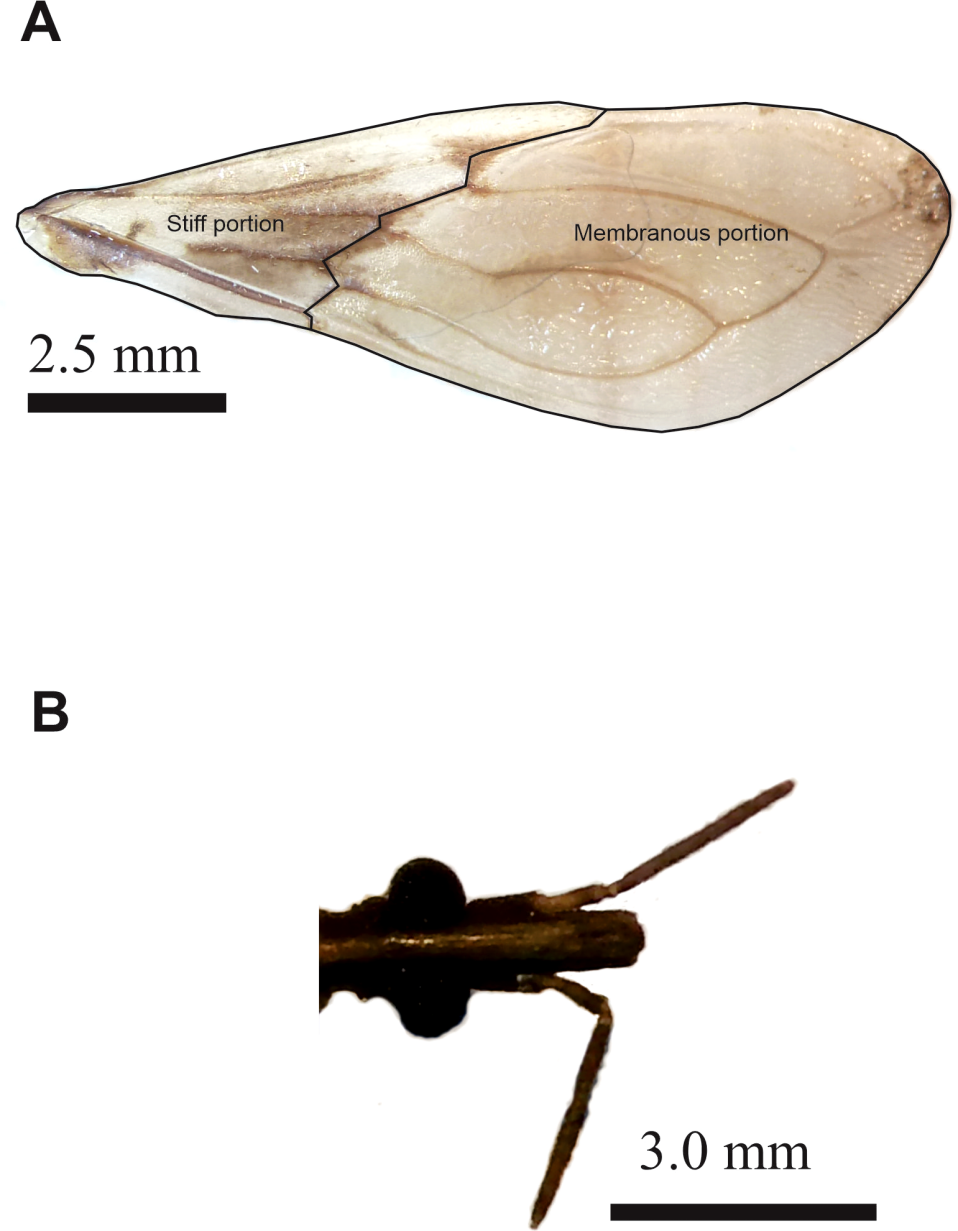
Photographs of the wing **(A)** and head **(B)** of *Triatoma garciabesi*, highlighting the different portions of the forewing. Details on the landmarks and semilandmarks used for morphometric analyzed are detailed in Verly et al. (26).

A total of 198 males from 24 populations were analyzed, each with a sample size of between 5 and 21 male specimens (**Table 1** 26). Digital images of the ventral view of the head and the dorsal view of the right forewing were captured to analyze the size and shape variables of flight-related traits, specifically the head and forewing structures (**Figure 2**). These traits were further characterized by assessing both stiff and membranous portions of the forewing, as outlined elsewhere [**Figure 2**, Verly et al. (26)]. All methodologies and techniques used for this study were previously explained in Verly et al. (26), which provides further details on the analytical framework and the population-specific data.

### Geographic and climatic variables and vegetation indexes

To obtain the geographic variables of each population, latitude, longitude, and altitude were recorded using Google Earth Pro. Climatic and vegetation data were extracted using ERAS 5 (Copernicus Climate Data Store) (31) at a spatial resolution of 28 × 28 km, covering a 10-year period (2013–2023). For this study, we included only variables considered influential in the spatial distribution of Triatominae or relevant to flight dispersal in insect dispersal (13, 25, 32–35).

The climatic variables considered were (1) annual mean temperature, (2) maximum temperature of the warmest month, (3) minimum temperature of the coldest month, (4) wind speed, (5) wind direction, (6) annual rainfall, and (7) relative humidity. Wind direction and velocity were extracted from the North–South and East–West components of winds. Relative humidity was calculated as: RH = 100 - 5 × (*T* − *T*_d_), where *T* is the average monthly temperature and *T*_d_ is the average monthly dew point temperature (36). The vegetation indexes included were (1) the Normalized Difference Vegetation Index (NDVI), (2) leaf area index low vegetation (LAILV), and (3) leaf area index high vegetation (LAIHV) (31). The leaf area index (LAI) quantifies the amount of leaf material in a canopy. By definition, it is the ratio of one-sided leaf area per unit ground area (31).

### Statistical analysis

Statistical analyses and results were first conducted by including all 24 populations in the study (i.e., species level of analysis) and then separately for the Eastern and Western lineage (i.e., lineages level of analysis). Each structure or part of the structure was analyzed separately. The landmark configurations were superimposed using a generalized Procrustes analysis (GPA) (37).

To test the populations for differences in the average size of the forewing, stiff, and membranous portions and head, we performed analysis of variance (ANOVA) followed by Tukey’s *post-hoc* tests. For these analyses, the software InfoStat version 2016 was used (38). To assess the degree of correlation between size and shape for each structure or part of structure, we fitted a multiple linear regression between the Procrustes coordinates and the centroid size. None of the structures showed an allometric effect, except for the membranous portion of the forewing that showed a significant allometric relationship (*p* = 0.0027), but with small allometric effects (*r* = 0.02). To test population differentiation for each structure or part of structure, a canonical variate analysis (CVA) was done. We calculated Procrustes distances between pairs of populations and evaluated their significance via a non-parametric test based on permutations (1,000 runs). These steps of analysis were done with MorphoJ version 1.07a (39). The Procrustes distances were represented in unrooted neighbor-joining (NJ) trees using the free software MEGA X 10.2.6 (40). We calculated bootstrap values for 1,000 replications following Ascarranuz et al. (41). The association of geographic distances with linear and geometric morphometric measurements of forewing, stiff, and membranous portions and head was analyzed using Mantel tests with the free software PASSAGE 2 2.0.11.6 (42). Morphological distance matrices between populations were constructed using Procrustes distances for shape measurements and Euclidean distances for size measurements. We tested the effect of geographic, climate, and vegetation cover on phenotypic changes of forewing, stiff, and membranous portions and head using a two-block partial least squares (PLS) analysis. PLS analysis tests for covariation between blocks of variables (shape or size of each structure within populations) were done using the Procrustes coordinates for shape or CS for size with geographic and climatic variables and vegetation indexes (43). To calculate a *p*-value and significance test, we generated 9,999 permutations for each comparison. PLS analyses were calculated using the R package *geomorph* (44) using the *two.b.pls* function. Additionally, to explore if there are associations between the NDVI, as a measure of habitat heterogeneity (45, 46), and shape variations in flight-related traits, we performed a Procrustes regression with permutations for each structure including the lineage and the populations nested within the lineage in the analysis. These analyses were conducted using the *procD.lm* function from the *geomorph* package in R.

## Results

### Differentiation of flight-related traits at the level of the distribution range of species and lineages

At the species distribution range level, the size component analysis revealed no significant differences in forewing size between populations [*F*_(197, 23)_ = 826.94, *p* = 0.9907]. However, the membranous and stiff portions of the forewing did show significant differences [*F*_(197, 23)_ = 8.00, *p* < 0.0001; *F*_(197, 23)_ = 9.55, *p* < 0.0001] (**Supplementary Material 1**). Head size did not show significant differences between populations [*F*_(174, 23)_ = 20.93, *p* = 0.1217].

**Figure 3:**
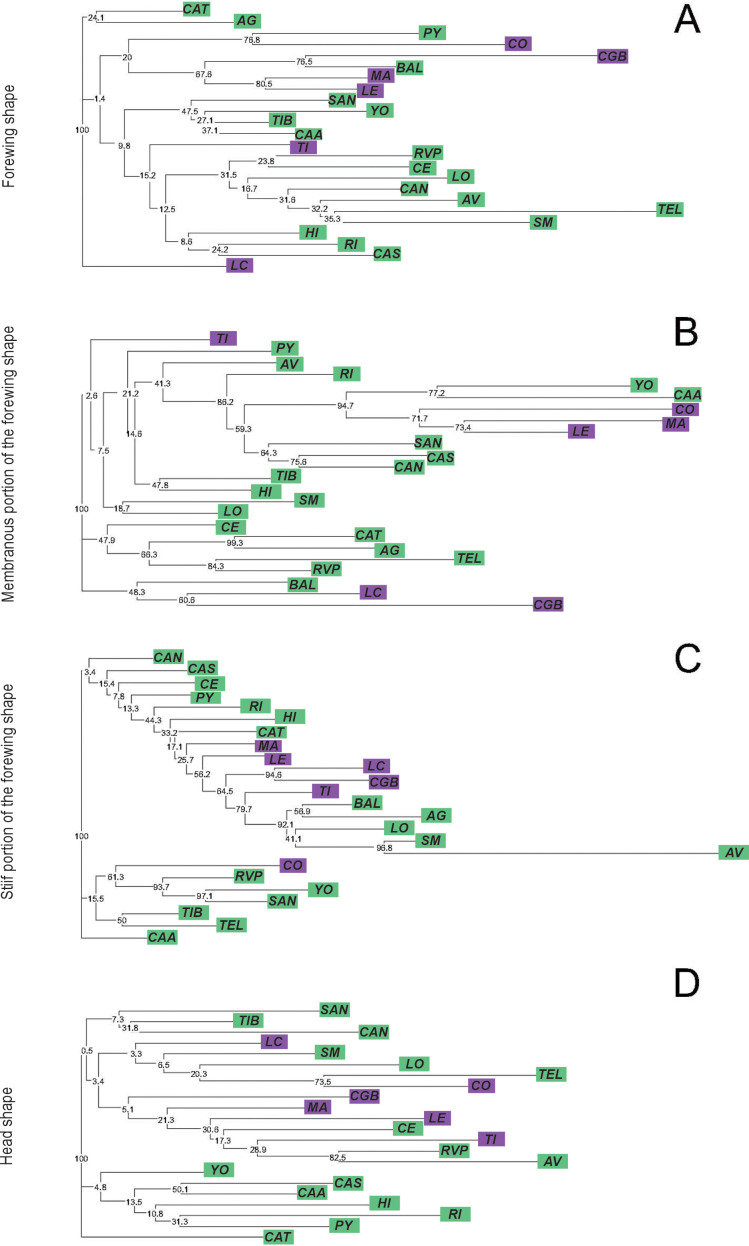
Neighbor-joining trees based on Procrustes distances between populations of *Triatoma garciabesi* performed from the forewing shape component **(A)** and the membranous **(B)** and stiff **(C)** portions of the forewing and head **(D)** for the species level distribution range of *T. garciabesi*. Numbers close to the nodes are 1,000 replicates of bootstrap values. Blue squares represent populations from the Eastern lineage and green squares denote populations from the Western lineage.

The ordination of the populations by CVA for forewing showed that the first and second canonical variables jointly explained 52.48% of the total variation (CV1: 34.31%, CV2: 18.17%). CGB was the only population with significantly different Procrustes distances compared to the others (**Supplementary Material 2**). The forewing NJ tree, based on Procrustes distances, revealed that only four nodes had bootstrap support greater than 70%. One of these nodes grouped together two populations from the Eastern lineage (MA and LE) (**Figure 3A**). For the membranous portion of the forewing, the CVA revealed that the first two axes explained 39.27% of the total variation (CV1: 24.65%, CV2: 14.62%). All pairwise Procrustes distances between populations were significantly different from zero (*p* < 0.0001, **Supplementary Material 2**). The NJ tree based on Procrustes distances indicated 8 from 21 nodes that were supported by bootstrap (values ≥ 70%). One of these nodes grouped two populations from the Eastern lineage, while five nodes grouped Western populations together (**Figure 3B**). Regarding the stiff portion of the forewing, the first two axes explained 45.03% (CV1: 28.00%, CV2: 17.03%). As with the membranous portion, all pairwise Procrustes distances between populations were significant (*p* < 0.0001, **Supplementary Material 2**). The NJ tree based on Procrustes distances showed that 7 out of 21 nodes were bootstrap-supported. One of these nodes grouped two populations from the Eastern lineage, while the other nodes grouped Western populations together (**Figure 3C**). For head, the first two discriminant factors from the CVA jointly explained 49.92% of the total variation (CV1: 28.42%, CV2: 21.50%). CGB had the highest number of significant Procrustes distances with 18 out of 21 pairwise comparisons (**Supplementary Material 2**). The NJ tree based on Procrustes distances indicated that 3 out of 20 clusters were supported by bootstrap values (**Figure 3D**).

**Figure 4:**
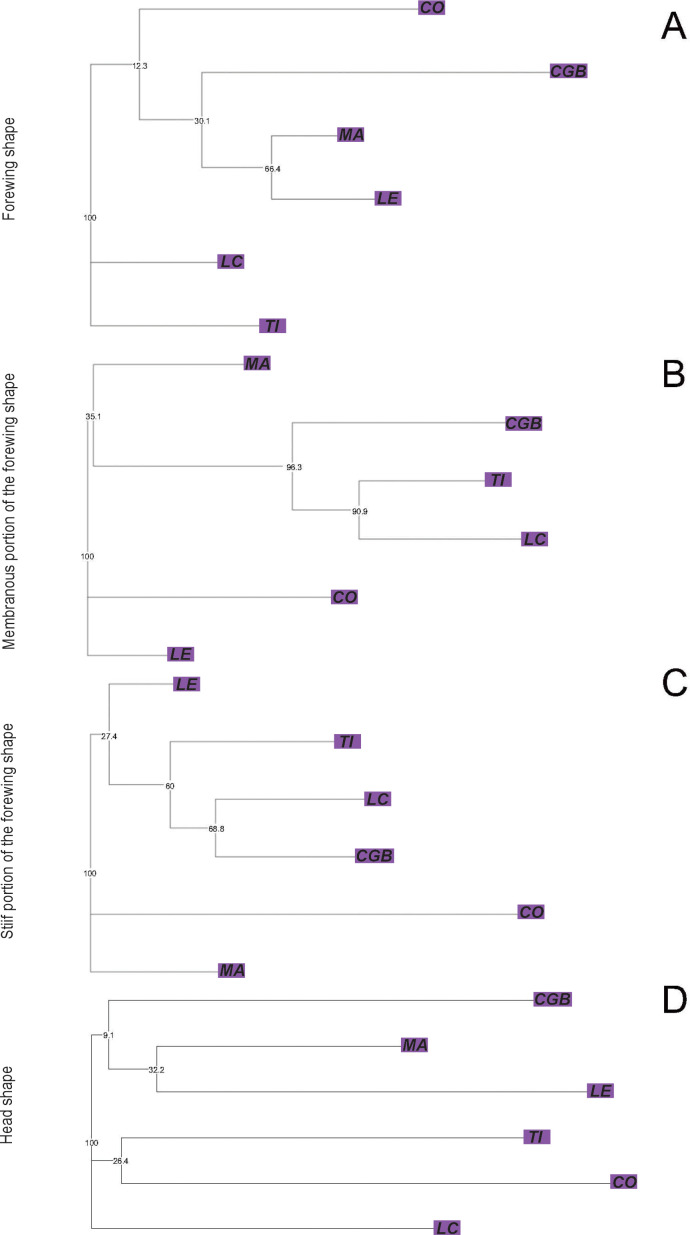
Neighbor-joining trees based on Procrustes distances between populations of *Triatoma garciabesi* performed from the forewing shape component **(A)** and the membranous **(B)** and stiff **(C)** portions of the forewing and head **(D)** for the Eastern lineage distribution range of *T. garciabesi*. Numbers close to the nodes are 1,000 replicates of bootstrap values.

The Eastern lineage showed significant differences in the size component of forewing [*F*_(52, 5)_ = 11.90, *p* < 0.0001], membranous, and stiff portions [*F*_(52, 5)_ = 16.73, *p* < 0.0001; *F*_(52, 5)_ = 12.37, *p* < 0.0001 for membranous and stiff portions, respectively] and head [*F*_(52, 5)_ = 6.44, *p* < 0.0001]. In all cases, CGB and CO exhibited the smallest structure or portion of structure significantly different from the other populations (Tukey *post-hoc* tests, *p* < 0.010) (**Supplementary Material 1**). The first two discriminant factors of the forewing CVA jointly explained 85.72% of the total variation (CV1: 62.81%, CV2: 22.91%). The forewing NJ tree based on Procrustes distances showed no bootstrap-supported clades (**Figure 4A**). For the membranous portion of the forewing, the first two CVA axes explained 82.68% of the total variance (CV1: 66.47%, CV2: 16.21%). All pairwise Procrustes distances between populations were significantly different from zero (*p* < 0.0001, **Supplementary Material 3**). The NJ tree based on Procrustes distances showed that three out of four clades were bootstrap-supported. One of the bootstrap-supported clades separated the CGB population from Formosa province from the TI and LC populations in Chaco province. Other bootstrap-supported clades grouped the TI and LC populations from Chaco province together (**Figure 4B**). For the stiff portion of the forewing, the first two axes explained 71.62% of the total variation (CV1: 55.83%, CV2: 15.79%). All pairwise Procrustes distances between populations were significantly different (*p* < 0.0001, **Supplementary Material 3**). The NJ tree based on Procrustes distances showed one out of four bootstrap-supported clades (**Figure 4C**). For the head, the first two axes of the CVA explained 81.03% of the total variation (CV1: 62.53%, CV2: 18.49%). Procrustes distances for the head from the LE, LC, and MA populations did not differ significantly from any of the other lineage populations (**Supplementary Material 3**). The NJ tree based on Procrustes distances did not show any bootstrap-supported clades (**Figure 4D**).

**Figure 5:**
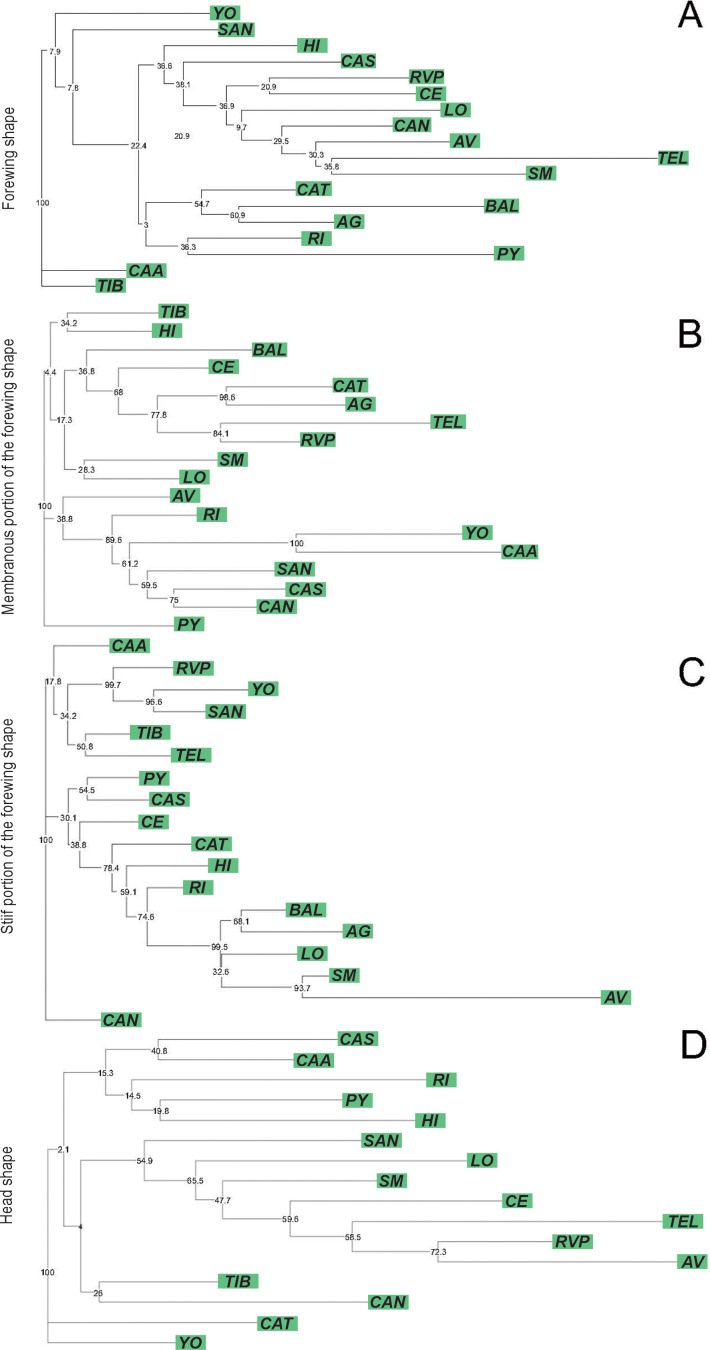
Neighbor-joining trees based on Procrustes distances between populations of *Triatoma garciabesi* performed from the forewing shape component **(A)** and the membranous **(B)** and stiff **(C)** portions of the forewing and head **(D)** for the Western lineage distribution range of *T. garciabesi*. Numbers close to the nodes are 1,000 replicates of bootstrap values.

The Western lineage showed no significant size differentiation for the forewing and head between populations [*F*_(125, 17)_ = 0.43, *p* = 0.9771; *F*_(125, 17)_ = 0.15, *p* = 0.9320 for forewing and head size, respectively]. However, significant differences were found for the size component of the membranous portion of the forewing [*F*_(125, 17)_ = 5.90, *p* < 0.0001]. The CE and AV populations had the smallest membranous portions, while HI, CAS, and CAA had the largest membranous portion (Tukey *post-hoc* tests, *p* < 0.01, **Supplementary Material 1**). The stiff portion of the forewing also showed significant differences across populations [*F*_(125, 17)_ = 8.25, *p* < 0.0001], where AG had the smallest stiff portion and PY, CAA, CAS, and YO populations had the largest ones (Tukey *post-hoc* tests, *p* < 0.01, **Supplementary Material 1**). For the shape component of forewing variation, the first two discriminant factors of the CVA explained 50.20% of the total variation (CV1: 36.01%, CV2: 14.19%). Most of the populations showed significant Procrustes distances (**Supplementary Material 4**), except for CAA, where 11 out of 17 Procrustes distance comparisons were not significant (**Supplementary Material 4**). The NJ forewing tree based on Procrustes distances showed no bootstrap-supported clades (**Figure 5A**). For the membranous portion of the forewing, the first two CVA axes explained 43.77% of the total variance (CV1: 26.80%, CV2: 16.80%). All pairwise Procrustes distances between populations showed significant differences (*p* < 0.0001, **Supplementary Material 3**). The NJ tree based on Procrustes distances showed that 7 out of 16 clades were bootstrap-supported. One of the bootstrap-supported clades separated the RI population from Salta province from five of the six Paraguayan populations (YO, CAA, SAN, CAS, and CAN). Additionally, clades grouping the Paraguayan populations YO and CAA, as well as CAS and CAN, were also supported by bootstrap values (**Figure 5B**). For the stiff portion of the forewing, the first two axes explained 43.97% of the total variation (CV1: 27.86%, CV2: 15.94%). All pairwise Procrustes distances between populations were significantly different (*p* < 0.0001, **Supplementary Material 3**). For this portion of the forewing, the NJ tree based on Procrustes distances shows that 7 out of 16 clades were bootstrap-supported. One of the bootstrap-supported clades separated the RVP population from La Rioja province from two of the six Paraguayan populations (YO and SAN). In addition, the clade with YO and SAN was supported by bootstrap values (**Figure 5C**). For the head, the first two CVA axes explained 55.83% of the total variation (CV1: 32.89%, CV2: 22.95%). Approximately 65% of the Procrustes distance comparisons between population pairs showed no significant differences (*p* > 0.050, **Supplementary Material 4**). The NJ tree based on these Procrustes distances showed 2 out of 14 bootstrap-supported clades (**Figure 5D**).

### Association between flight-related traits and geographical distances at the level of the distribution range of species and lineages

At the species, Eastern, and Western distribution levels, the Mantel tests revealed positive and significant association between shape of all flight-related traits and geographic distances (**Table 2**).

**Table 2:** Results of Mantel tests to evaluate the correlation between the shape and size components of forewing, membranous and stiff portions of forewing and head with geographic distances at the species level and lineages distribution ranges for *Triatoma garciabesi*.

Structure	Component	Species level		Lineage based on *coI*			
				Eastern		Western	
		*r*	*P*-value	*r*	*P*-value	*r*	*P*-value
Forewing	Shape	0.30	2.00x10^-5^ ***	0.46	9.23x10^-3^ **	0.34	1.00x10^-5^ ***
	Size	0.06	0.24	0.42	0.02*	0.02	0.30
Stiff portion	Shape	0.04	0.01*	0.67	5.80x10^-3^ **	0.01	0.02*
	Size	0.42	1.22x10^-3^ **	0.38	0.02*	0.36	5.22x10^-3^ **
Membranous portion	Shape	0.23	1.30x10^-4^ ***	0.31	0.01*	0.40	1.00x10^-5^ ***
	Size	0.11	2.43x10^-3^ **	0.47	8.13x10^-3^ **	0.21	6.30x10^-4^ ***
Head	Shape	0.32	1.00x10^-5^ ***	0.70	6.33x10^-3^ **	0.36	1.00x10^-5^ ***
	Size	0.42	1.55x10^-3^ **	0.04	0.09	0.31	9.44x10^4^ **

*P < 0.05, **P < 0.01, ***P < 0.001.

The correlation coefficient (*r*) and the *P*-value were presented. All tests were performed with 10,000 permutations.

At the species distribution level, forewing size showed no significant association with geographic distances. However, the Mantel test showed a positive and significant association for the stiff portion, membranous portion, and head (**Table 2**). For the Eastern lineage, no significant association was found between head size and geographic distances. However, a positive and significant association was observed for forewing, stiff, and membranous portions. In contrast, for the Western lineage, forewing size did not show significant results. Nonetheless, the Mantel test revealed positive and significant associations for the stiff portions and membranous portions and head (**Table 2**).

### Association between flight-related traits with geographic and climatic variables and vegetation indexes

**Figure 6:**
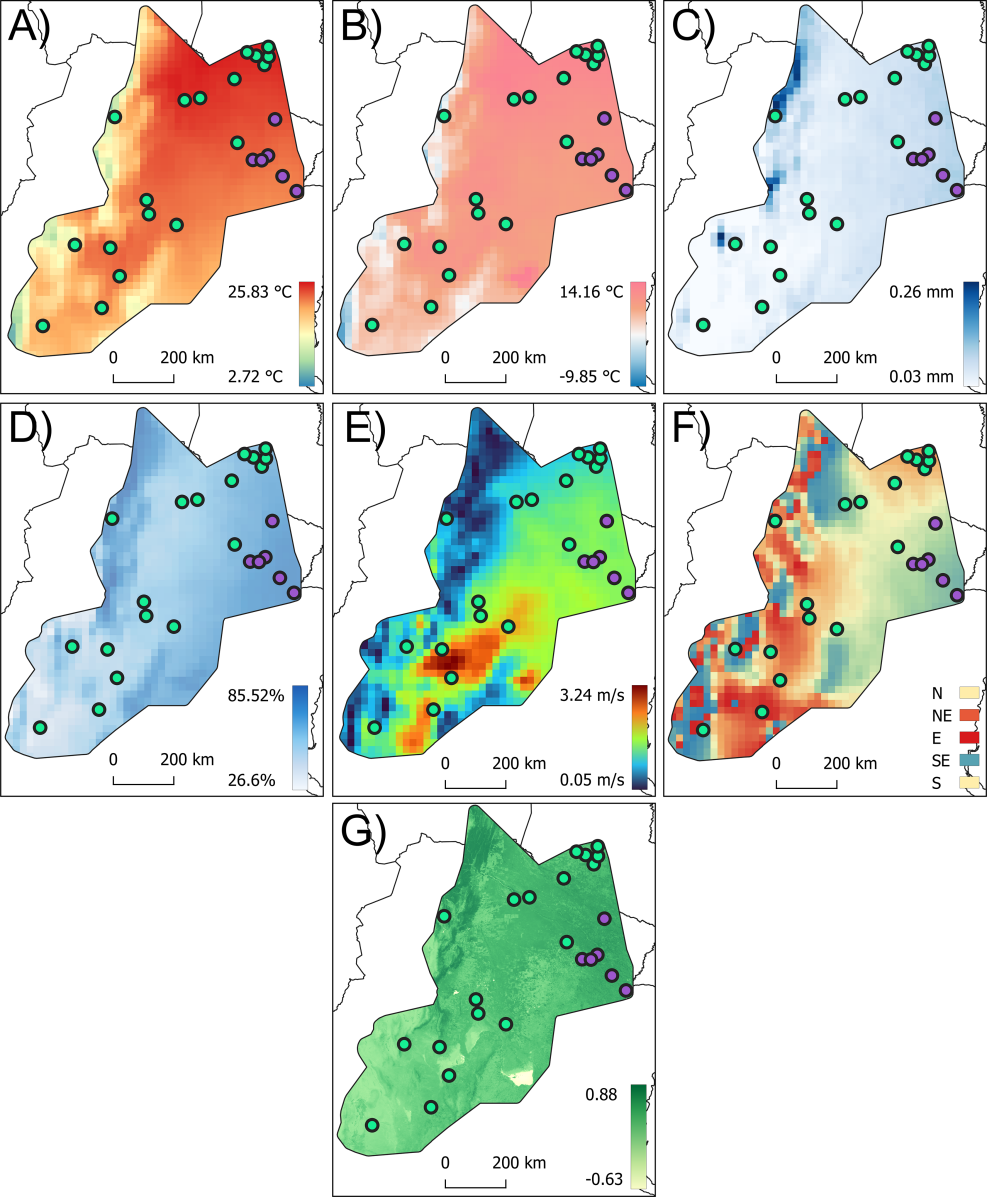
Variation of climatic and vegetation cover index (NDVI) across the distribution area of *Triatoma garciabesi*. The variables represented in these maps best explain the variation in flight-related traits: annual mean temperature **(A)**, minimum temperature of the coldest month **(B)**, annual rainfall **(C)**, relative humidity **(D)**, wind speed **(E)**, wind direction **(F)**, and NDVI **(G)**.

At the species distribution level, the results of the PLS analyses for all flight-related traits revealed significant correlations with the PLS1, which accounted for, in all cases, 100% of the covariation (**Supplementary Material 5**). For the forewing size component, PLS1 was primarily associated with relative humidity (*r* = −0.53) and wind speed (*r* = 0.44) (**Figures 6D, E**, **Supplementary Material 5**). For the membranous portion size, PLS1 was predominantly correlated with relative humidity (*r* = −0.51) and wind speed (*r* = 0.41), while for the stiff portion size, PLS1 was mainly correlated with relative humidity (*r* = −0.44), wind speed (*r* = 0.39), and annual rainfall (*r* = −0.38) (**Figures 6C–E**, **Supplementary Material 5**). For head size, PLS1 was mainly correlated with latitude (*r* = −0.47) and wind speed (*r* = 0.40) (**Figure 6E**, **Supplementary Material 5**). At the Eastern lineage populations, PLS analyses for the size of all flight-related traits revealed significant correlations with the PLS1, which accounted for, in all cases, 100% of the covariation (**Supplementary Material 5**). For forewing size, PLS was primarily associated with wind direction (*r* = 0.41) (**Figure 6F**) and the minimum temperature of the coldest month (*r* = −0.39) (**Figure 6B**). For the size of the membranous portion, PLS1 was primarily correlated with the minimum temperature of the coldest month (*r* = −0.41) (**Figure 5B**) and wind direction (*r* = 0.40) (**Figure 6F**). For the size of the stiff portion, PLS1 was mainly associated with latitude (*r* = −0.48), annual rainfall (*r* = −0.40) (**Figure 6C**), and wind speed (*r* = 0.37) (**Figure 6E**). For head size, PLS1 was mainly correlated with wind direction (*r* = 0.40) (**Figure 6F**), relative humidity (*r* = 0.37) (**Figure 6D**), and latitude (*r* = −0.37) (**Supplementary Material 5**). For the Western lineage populations, the size of all flight-related traits revealed significant correlations with the PLS1 accounting for 100% of the covariation (**Supplementary Material 5**). For the forewing size, PLS1 was mainly correlated with latitude (*r* = −0.49) and relative humidity (*r* = −0.43) (**Figure 6D**). The membranous portion of the forewing was mainly associated with annual rainfall (*r* = −0.46) (**Figure 6C**), latitude (*r* = −0.46), and relative humidity (*r* = −0.42) (**Figure 6D**, **Supplementary Material 5**). For the stiff portion, PLS1 was mainly correlated with latitude (*r* = −0.48) and annual rainfall (*r* = −0.40) (**Figure 6C**). In the case of head size, PLS1 was primarily correlated with latitude (*r* = −0.49), longitude (*r* = −0.38), and minimum temperature of the coldest month (*r* = −0.38) (**Figure 6B**, **Supplementary Material 5**).

PLS analyses for the forewing shape components across the species distribution level showed a significant correlation. PLS1 explained 79.31% of the total covariation and was mainly correlated with the NDVI (*r* = −0.37) and LAILV (*r* = 0.38) indexes (**Figure 6G**, **Supplementary Material 6**). The membranous portion of the forewing shape also showed a significant correlation, with PLS1 explaining 78.86% of the total covariation and was mainly correlated with longitude (*r* = −0.46) and latitude (*r* = −0.36) (**Supplementary Material 6**). A similar trend was observed for the stiff portion of the forewing, where PLS1 explained 61.92% of the total covariation, with significant correlations with wind speed (*r* = −0.44) (**Figure 6E**) and latitude (*r* = 0.39), among others (**Supplementary Material 6**). For head shape, PLS analysis also indicated a significant correlation, with PLS1 explaining 77.04% of the total covariation. This PLS was mainly correlated with latitude (*r* = 0.50), the minimum temperature of the coldest month (*r* = 0.46), and annual mean temperature (*r* = 0.40) (**Figures 6A, B**, **Supplementary Material 6**). At the Eastern lineage distribution range, forewing shape showed significant covariation with geographic, climatic variables, and vegetation indexes. PLS1 explained 87.54% of the total covariation and was mainly explained by wind direction (*r* = −0.39), annual mean temperature (*r* = −0.36), and latitude (*r* = 0.35) (**Figures 6A, F**, **Supplementary Material 6**). The membranous portion of the forewing shape exhibited a significant correlation, with PLS1 explaining 86.18% of the total covariation, primarily associated with latitude (*r* = 0.40), annual mean temperature (*r* = −0.36) (**Figure 6A**), and altitude (*r* = 0.37). For the stiff portion of the forewing shape, PLS1 explained 77.01% of the total covariation and was mainly correlated with annual mean temperature (*r* = −0.40) (**Figure 6A**), among others (**Supplementary Material 6**).

Head shape PLS also indicated significant correlation; PLS1 explained 75.45% of the total covariation and was mainly correlated with wind direction (*r* = −0.39) (**Figure 6F**) and latitude (*r* = 0.36). For the Western lineage distribution range, forewing shape showed significant PLS correlation; PLS1 explained 83.36% of the covariation and was mainly correlated with minimum temperature of the coldest month (*r* = 0.41) (**Figure 6B**), latitude (*r* = −0.39), and longitude (*r* = 0.37). The membranous and stiff portion of the forewing shape also showed significant PLS correlations. PLS1 explained 84.07% and 78.64% for membranous and stiff portions, respectively, and was mainly correlated with annual rainfall (*r* = −0.46) (**Figure 6C**), latitude (*r* = −0.46), and relative humidity (*r* = −0.42) (**Figure 6D**). The stiff portion was mainly correlated with wind speed (*r* = −0.44) (**Figure 6E**) and latitude (*r* = 0.41). Head shape also showed significant covariation in the PLS analysis. PLS1 retained 84.97% of the total covariation and was mainly correlated with latitude (*r* = −0.46) and with the minimum temperature of the coldest month (*r* = 0.45) (**Figure 6B**) among others (**Supplementary Material 6**).

Results of the Procrustes regression performed with the shape component of each flight-related trait with the NDVI variation (**Figure 6G**), lineage, and population nested within the lineage showed significant effects for all components (*p* < 0.05). However, the correlation coefficients were low in all cases and no significant effect was found for lineage in the membranous portion of the forewing (**Table 3**).

**Table 3:** Results of the permutation analysis of variance (ANOVA) for the variation in the shape component of the flight-related traits measured for *Triatoma garciabesi*.

Structure	Component of the model	df	SS	Rsq	*F*	*Z*	*P*-value
Forewing	NDVI	1	0.003	0.009	2.523	2.070	0.019
	Lineage	1	0.003	0.008	2.442	2.033	0.021
	Population	21	0.096	0.295	4.093	11.224	0.000
	Residuals	165	0.185	0.571			
	Total	168	0.324				
Membranous portion	NDVI	1	0.004	0.025	5.894	3.007	0.000
	Lineage	1	0.001	0.008	1.893	1.396	0.088
	Population	21	0.039	0.218	2.474	5.872	0.000
	Residuals	168	0.127	0.706			
	Total	191	0.180				
Stiff portion	NDVI	1	0.023	0.038	11.933	4.091	0.000
	Lineage	1	0.005	0.008	2.408	1.908	0.029
	Population	21	0.256	0.430	6.438	8.698	0.000
	Residuals	165	0.312	0.524			
	Total	188	0.595				
Head	NDVI	1	0.004	0.017	3.313	2.530	0.007
	Lineage	1	0.004	0.016	3.255	2.587	0.005
	Population	19	0.048	0.213	2.237	5.681	0.000
	Residuals	145	0.163	0.726			
	Total	166	0.224				

df, Degrees of Freedom; SS, Sum of Squares; Rsq, R-Squared; F, F-statistic; Z, Z-score.

The models include the Normalized Difference Vegetation Index (NDVI), lineage and nested localities within the lineages.

## Discussion

Our study documents significant differences in the size component of both the membranous and stiff portions of the forewings across the distribution range of *T. garciabesi*, with a decoupling between these two portions. Populations do not exhibit both portions in comparably similar sizes; populations with smaller membranous portions are not the same as those with smaller stiff portions, and *vice versa*. While no significant shape differences (Procrustes distances) were observed across all population pairs for the forewing and head, significant differences were detected in the membranous and stiff portions. Specifically, the Eastern lineage showed differences in the size component of all flight-related traits, while shape differences followed a trend similar to that at the species level. In terms of the shape component of flight-related traits, there was a strong and consistent association between phenotypic and geographic distances across all levels of comparison (species and lineage ranges). However, for the size component, no association was observed between the forewing size and geographic distance at the species level or for the Western lineage, although the membranous and stiff portions showed significant geographic variation. Geographic, climatic, and vegetation indexes explained the covariation with flight-related traits, with relative humidity, wind speed, and latitude being the most significant environmental factors affecting the size component of flight traits. Wind direction, annual rainfall, and the minimum temperature of the coldest month also contributed, but to a lesser extent. For the shape component, latitude was the strongest covariate, followed by mean annual temperature, minimum temperature of the coldest month, wind speed and direction, and other factors such as NDVI, LAILV, annual rainfall, relative humidity, and altitude. Notably, NDVI, as a measure of habitat heterogeneity, showed a strong association with the variation in the shape of flight-related traits.

The differentiation of the size component of morphometric traits across populations has been widely recognized as strongly influenced by ecological factors (47–49). The decoupling between the membranous and stiff portions observed in both species and lineage ranges may be linked to the functional differences of these wing portions during dispersal. The stiff portion of the forewing plays a crucial role in supporting and regulating wing movements, while the membranous portion is more flexible and subject to deformation by aerodynamic forces during flight (50). Although the specific contributions of these forces to wing deformation are not yet fully understood, it is clear that a larger membranous portion can be deformed more easily by aerodynamic and inertial forces, improving flight performance (14).

For *T. garciabesi*, this variation and decoupling of forewing portions could be related to the ecological diversity exhibited by different populations. This species mainly occupies sylvatic habitats, being a bird-associated arboreal species that inhabits the loose bark of trees at high population densities throughout the year (9). In particular, in the Telteca Natural and Cultural Reserve located in the Monte Desert ecoregion of Argentina, Carbajal-de-la-Fuente et al. (51) found *T. garciabesi* adults, mostly males, on the loose bark of *Prosopis flexuosa* in the spring. This species frequently invades rural houses during the warmer seasons, occupying peridomestic habitats, mostly associated with chicken coops (6, 7, 9). Moreover, this species shows higher densities in less anthropized landscapes (19). Variation in landscape characteristics of the different localities where this species was collected could be related to the morphometric differentiation across flight-related traits observed in this study. Shape variation in flight-related traits was consistently observed for the membranous and stiff portions of the forewings at the different levels of comparison. Spatial variation in these attributes could be characterized by differences in selective pressures exerted in different habitats, which may relate to the flight behavior characteristics of each population. Triatomines are univoltine insects that have only one generation per year (52). Living in stable habitats should drive the evolution of dispersal towards lower rates, whereas living in habitats with a rapid turnover rate is expected to stimulate the evolution of high dispersal capacities (53). Adults of *T. garciabesi* fly during warm periods, mainly in late spring, from sylvatic environments and/or peridomestic structures (7).

Our results showed that the shape of flight-related traits had a significant association with geographic distance, implying that populations may be structured with reduced exchange between distant populations. The shape component of morphometric traits across populations has been suggested to be influenced by genetic characteristics (49). When habitats become difficult to traverse or there is a decrease in the fitness of dispersing individuals (maladaptation to new environmental conditions), the gene flow is reduced and the population is structured locally. Cavallo et al. (7) showed that *T. garciabesi* did not aggregate at the locality level, but it was more frequently found near houses (≤100 m) with intermediate vegetation cover (NDVI values between 0.25 and 0.35) and in areas with intermediate vegetation cover (NDVI between 0.3 and 0.4) within a 1,000-m radius around the house. This study also found that the shape of flight-related traits correlated with NDVI, reinforcing the idea that vegetation cover and landscape heterogeneity play a significant role in shaping the morphology and dispersal capacity of *T. garciabesi*. These results, along with those reported by Cavallo et al. (7) and Fiad et al. (19), suggest that *T. garciabesi* is sensitive to vegetation cover and landscape features, likely promoting a disaggregated distribution mosaic, isolation by distance, and limited gene exchange between distant populations. Although the NDVI does not measure habitat conservation or the type of vegetation cover, it is an alternative used as a measure of habitat heterogeneity, and fluctuation in flight-related traits across populations has been sensitive to its variation (54). Additionally, the sensitivity of *T. garciabesi* to vegetation cover and preserved landscapes is linked to its bird hosts. Higher bird species richness, which is associated with higher biomass and temperature values, has been observed in less anthropized areas of the Argentine Dry Chaco Forest (55).

Wing morphology has been shown to respond to environmental variation in other insect species (e.g. 25, 56, 57). This observation has been made particularly during development when the genes that should be activated for wing development are altered by environmental factors (56). *T. garciabesi* is likely to remain in the same habitat during the nymphal period until adults finally disperse by flight, which holds true for this species as well. The association of variation in body size, forewing size, and/or shape with latitude has also been found in other insect species including other *Triatoma* species (*T. guasayana* and *T. infestans*) (e.g., 13, 25, 58, 59). The way in which latitude is associated with the forewing morphometric variation or other related traits is variable in insects. In particular, for *T. infestans*, forewing size and latitude show a positive relationship (Bergmann’s rule) (25). For *T. guasayana*, forewing shape but not size was associated with latitude (13). Our results showed a consistently negative association of all flight-related trait size with latitude (inverse Bergmann’s rule), and a variable positive or negative association of flight-related trait shape with latitude. Wind speed and direction variation (see **Figures 6E**) were also associated with the size and/or shape of the membranous and stiff parts of the forewing and head. When there was a positive covariation, it means that the increase in the size of the forewing was accompanied by an increase in the stiff or membranous portions and/or in the size of the head. For wind direction, a positive association suggests that forewing size increased as the wind shifts from north to south and east to west. This result suggests that wind speed and direction may influence forewing and head variation in populations of *T. garciabesi*, an environmental feature poorly studied in triatomines but with the potential to be an important determinant of species distribution, movements, and dispersal at different scales, as observed in another insect vector (60). One of the most important drivers of phenotypic plasticity in insects is temperature (e.g., 61). For *T. garciabesi*, our data suggested that shape variation and, to a lesser extent, size variation of flight-related traits were sensitive to the included temperature-related traits (mean annual temperature and minimum temperature of the coldest month variation; see **Figures 6A**). Temperature was found to influence size variation in insects. The temperature–size rule suggests that insects developed at high temperatures have smaller sizes (62). Our findings revealed consistency with Atkinson’s (62) temperature–size rule. Temperature also affects wing loading (63), which depends on wing shape. Thus, we would expect populations from cooler areas with a minimum temperature of the coldest month to have reduced wing loading to compensate for reduced flight performance due to smaller adult size (64). Reduced wing loading is achieved by increasing wing area (64). Populations of *T. garciabesi* from colder climates (see **Figures 6A**) had short and thin forewings, consistent with the forewing loading prediction and thus an aerodynamic hypothesis. Relative humidity is an environmental factor that can affect various aspects of insect life (65). In *T. infestans*, both nymphs and adults prefer to remain at approximately 0% RH, regardless of their nutritional status, and females showed a preference for oviposition at low RH (66). Variation in the size component of flight-related traits showed a negative relationship with relative humidity variation (see **Figure 6D**) in almost all cases, implying the small size of flight-related traits at high relative humidity. High relative humidity was associated with tolerance to prolonged fasting; conversely, low relative humidity would not tolerate prolonged starvation.

While our study offers important insights into the ecological and environmental influences on the morphology of *T. garciabesi*, it is observational in nature and cannot identify the specific mechanisms underlying the flight-dispersive process. Although the distribution range of the Eastern lineage is geographically restricted compared to the Western lineage, the sample imbalance may influence the interpretation of our results. Future research should aim to balance the sample sizes across lineages for a more robust analysis. While our current study focused primarily on interpopulation variation, examining intrapopulation differences could provide additional insights into the factors influencing trait variation. Experimental studies are needed to further investigate the relative importance of each flight-related trait in dispersal and to explore the precise mechanisms by which environmental factors influence wing morphology and dispersal behavior.

## Conclusion

This study provides compelling evidence of the influence of climatic, geographic, and vegetation factors on flight-related traits in *T. garciabesi*. The species appears to be sensitive to vegetation cover and landscape features, and we highlight significant variation in flight-related traits across the species’ distribution range, with signs of isolation by distance. Our results underscore the role of environmental factors—particularly temperature, humidity, and vegetation cover—in shaping the morphology and dispersal patterns of *T. garciabesi*, offering important insights into the species’ ecological adaptation and potential dispersal dynamics.

## Data Availability

The original contributions presented in the study are included in the article/**Supplementary Material**. Further inquiries can be directed to the corresponding authors.

## Supplementary material

Supplementary Material 1 Box plot (median and standard deviation) of the size component (centroid size) of the membranous portion of the forewing **(A)**, stiff portion of the forewing **(B)** across the species-level distribution range of *Triatoma garciabesi*. Forewing **(C)**, membranous portion of forewing **(D)**, stiff portion of forewing **(E)**, head **(F)** of the Eastern lineage and membranous **(G)** and stiff portions of forewing **(H)** of the Western lineage are also shown. The solid horizontal line represents the median. Different letters above boxes indicate statistical difference at *P* < 0.05 (Tukey tests). Population codes as in **Table 1**.

Supplementary Material 2 Matrices of pairwise Procrustes distances and pairwise permutation tests among all pairs of populations for the shape components of forewing, membranous and stiff portions of the forewing and head for the species-level distribution range of *Triatoma garciabesi.* Population codes as in **Table 1**.

Supplementary Material 3 Matrices of pairwise Procrustes distances and pairwise permutation tests among all pairs of populations for the shape components of forewing, membranous and stiff portions of the forewing and head for the Eastern lineage distribution range of *Triatoma garciabesi*. Numbers close to the nodes are 1,000 replicates of bootstrap values.

Supplementary Material 4 Matrices of pairwise Procrustes distances and pairwise permutation tests among all pairs of populations for the shape components of forewing, membranous and stiff portions of the forewing and head for the Western lineage distribution range of *Triatoma garciabesi*. Numbers close to the nodes are 1,000 replicates of bootstrap values.

Supplementary Material 5 Partial Least Square results between the size component of: forewing, membranous portion of the forewing, stiff portion of the forewing and head with geographic and climatic variables and vegetation indexes. Geographic variables: LAT, latitude, LONG, longitude and ALT, altitude. Climatic variables, TM, annual mean temperature, TMMMC, maximum temperature of the warmest month, TMMMF, minimum temperature of the coldest month, VELOC, wind velocity, DV, wind direction, PREC, annual rainfall and RH, relative humidity. Vegetation indexes, NDVI, Normalized Difference Vegetation Index, LAILV, leaf area index low vegetation and LAIHV, leaf area index high vegetation.

Supplementary Material 6 Partial Least Square results between the shape component of: forewing, membranous portion of the forewing, stiff portion of the forewing and head with geographic and climatic variables and vegetation indexes. Geographic variables: LAT, latitude, LONG, longitude and ALT, altitude. Climatic variables, TM, annual mean temperature, TMMMC, maximum temperature of the warmest month, TMMMF, minimum temperature of the coldest month, VELOC, wind velocity, DV, wind direction, PREC, annual rainfall and RH, relative humidity. Vegetation indexes, NDVI, Normalized Difference Vegetation Index, LAILV, leaf area index low vegetation and LAIHV, leaf area index high vegetation.
