# Supplementary figures and images for: Bug off or bug out: mapping flight secrets of Triatoma garciabesi (Hemiptera: Reduviidae) through climate, geography, and greenery
Source: Front Insect Sci. 2025 Jan 28;5:1532298. doi: 10.3389/finsc.2025.1532298 (PMC11810922; doi:10.3389/finsc.2025.1532298)

A

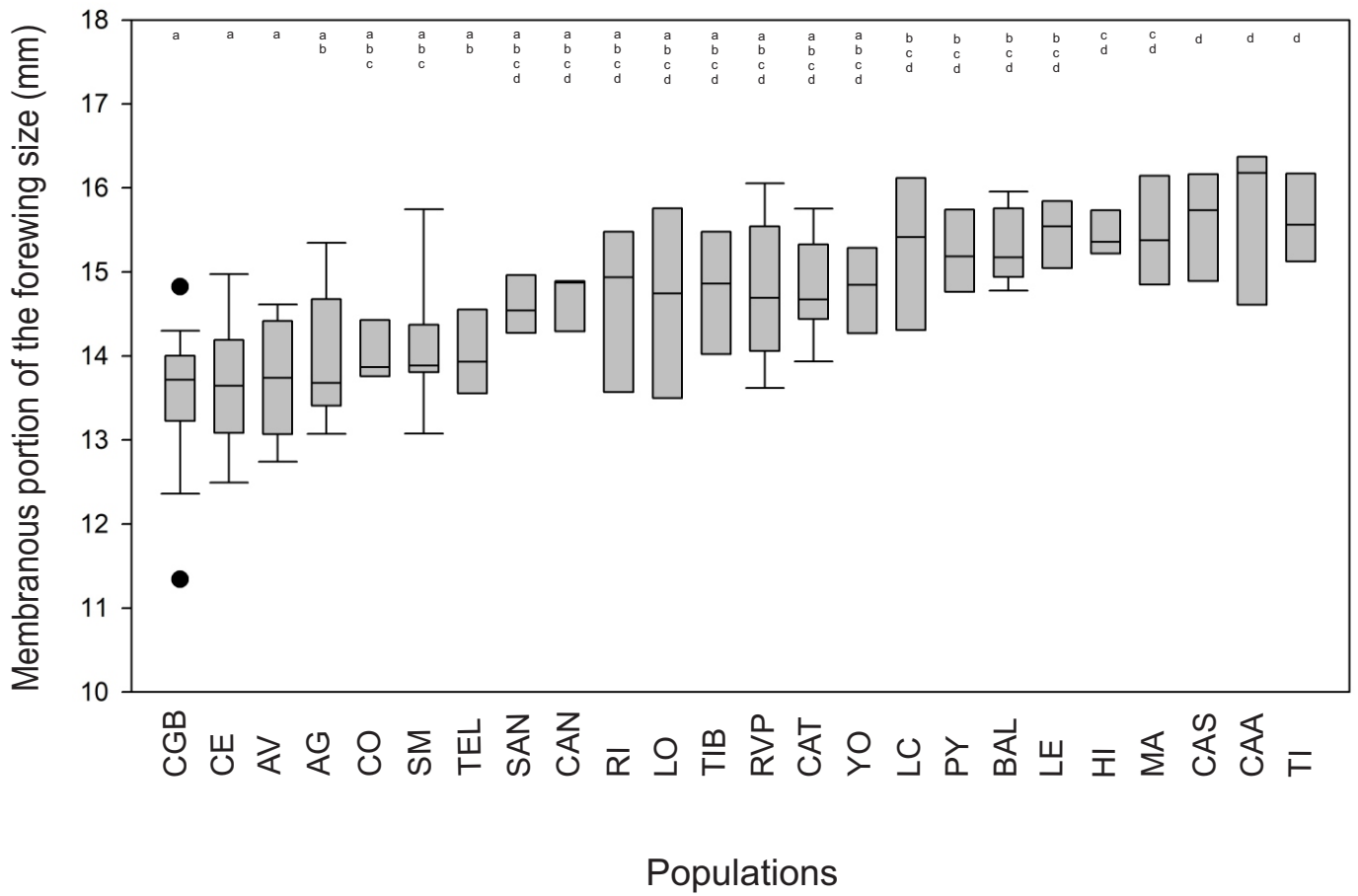

B

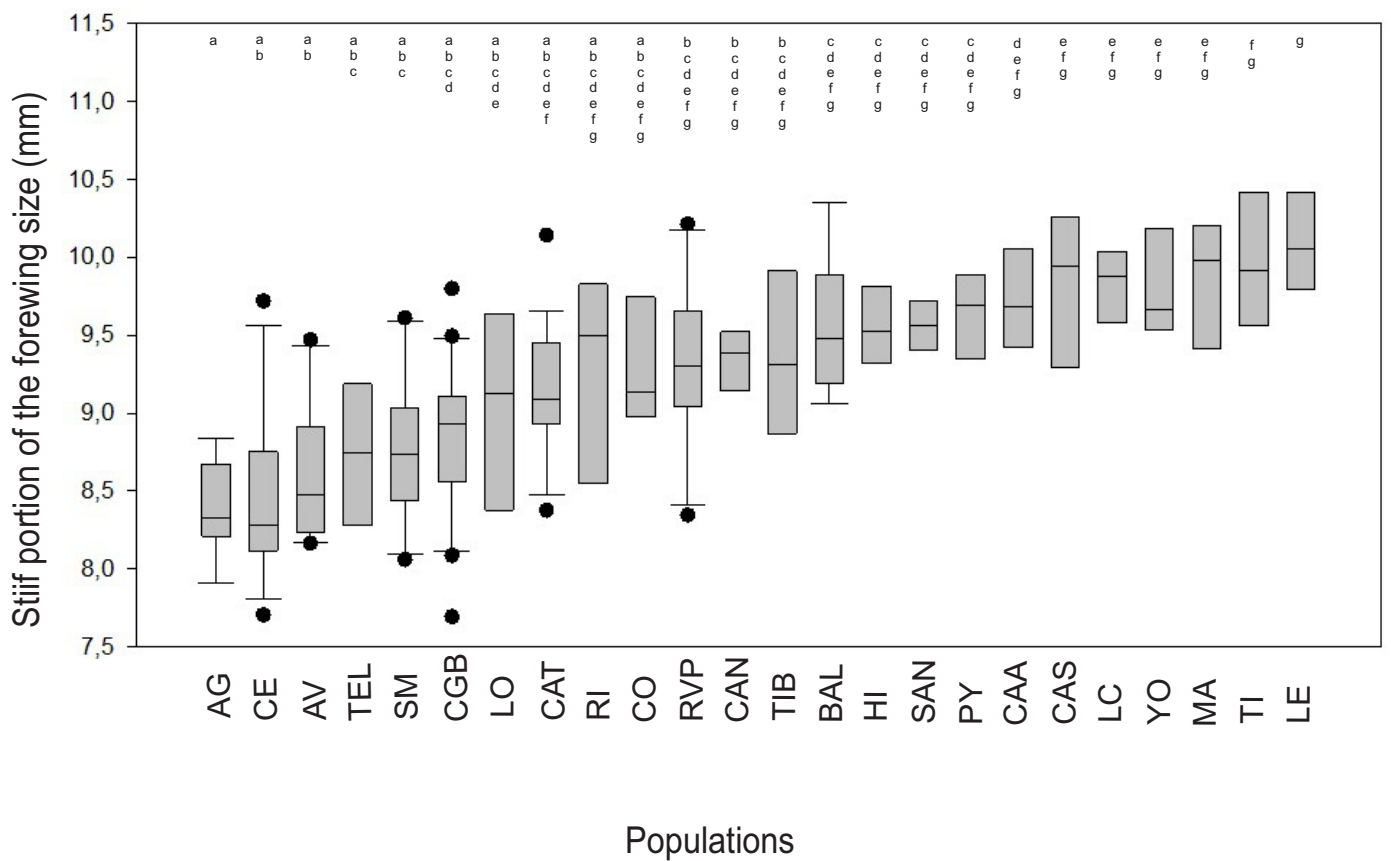

C

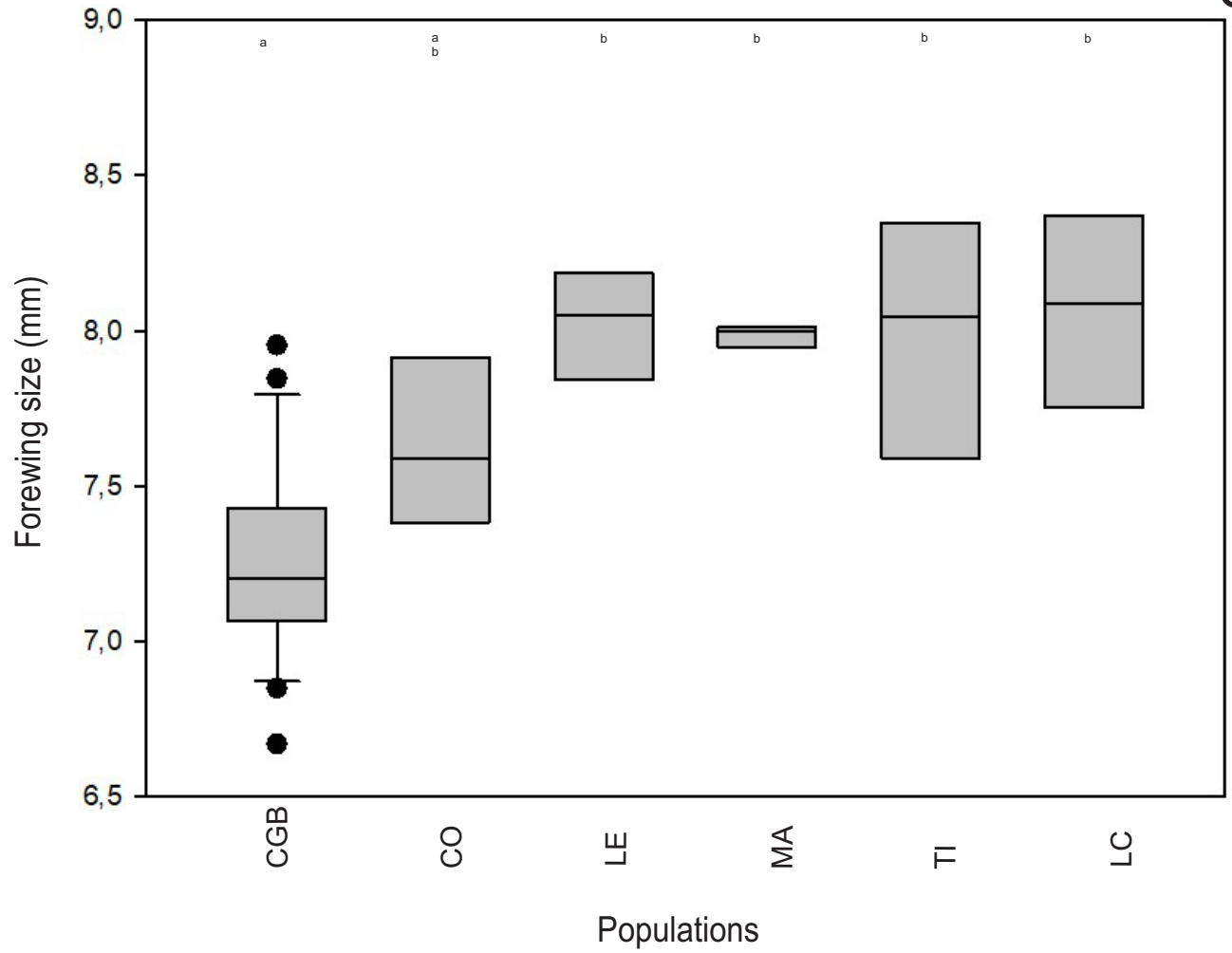

D

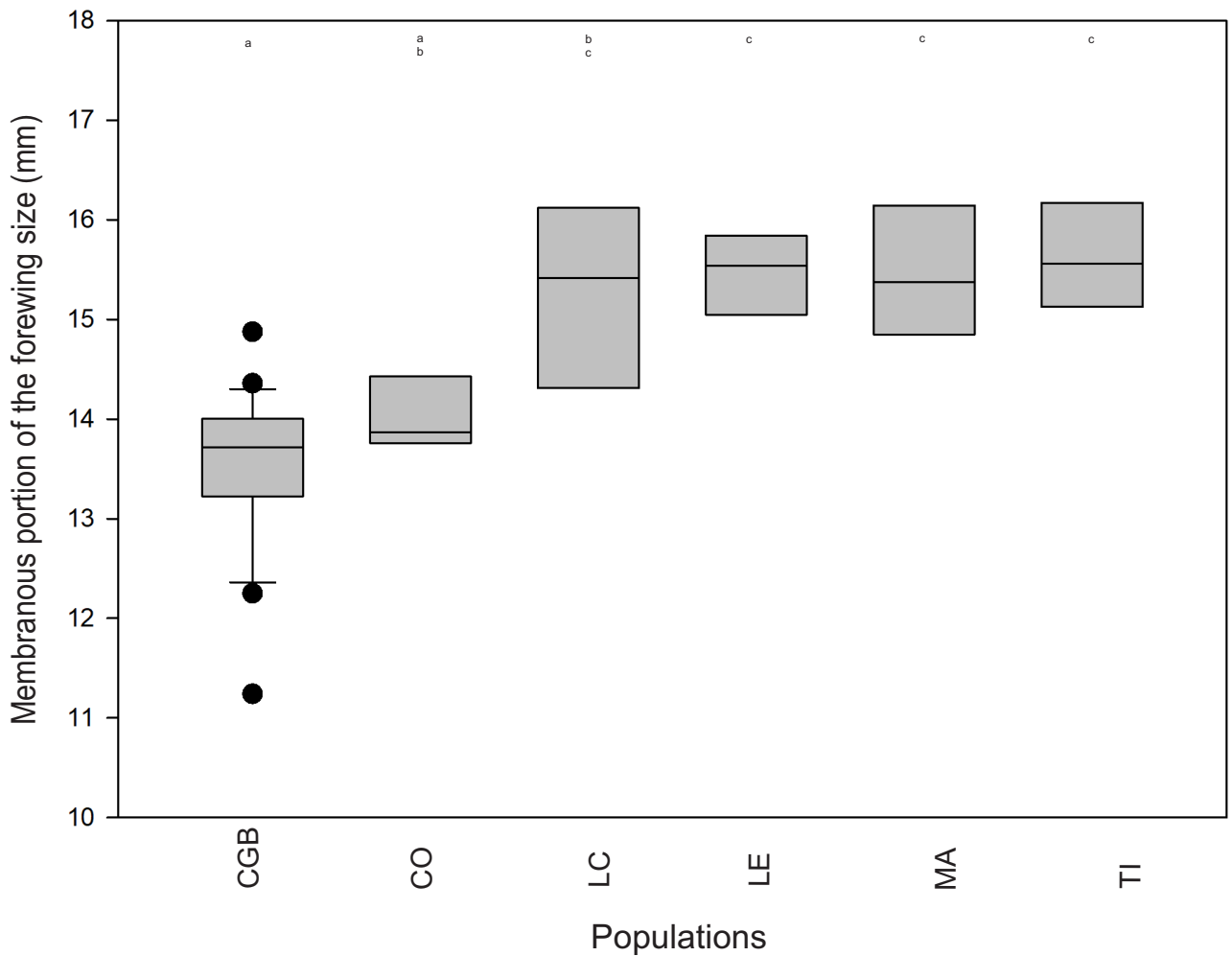

E

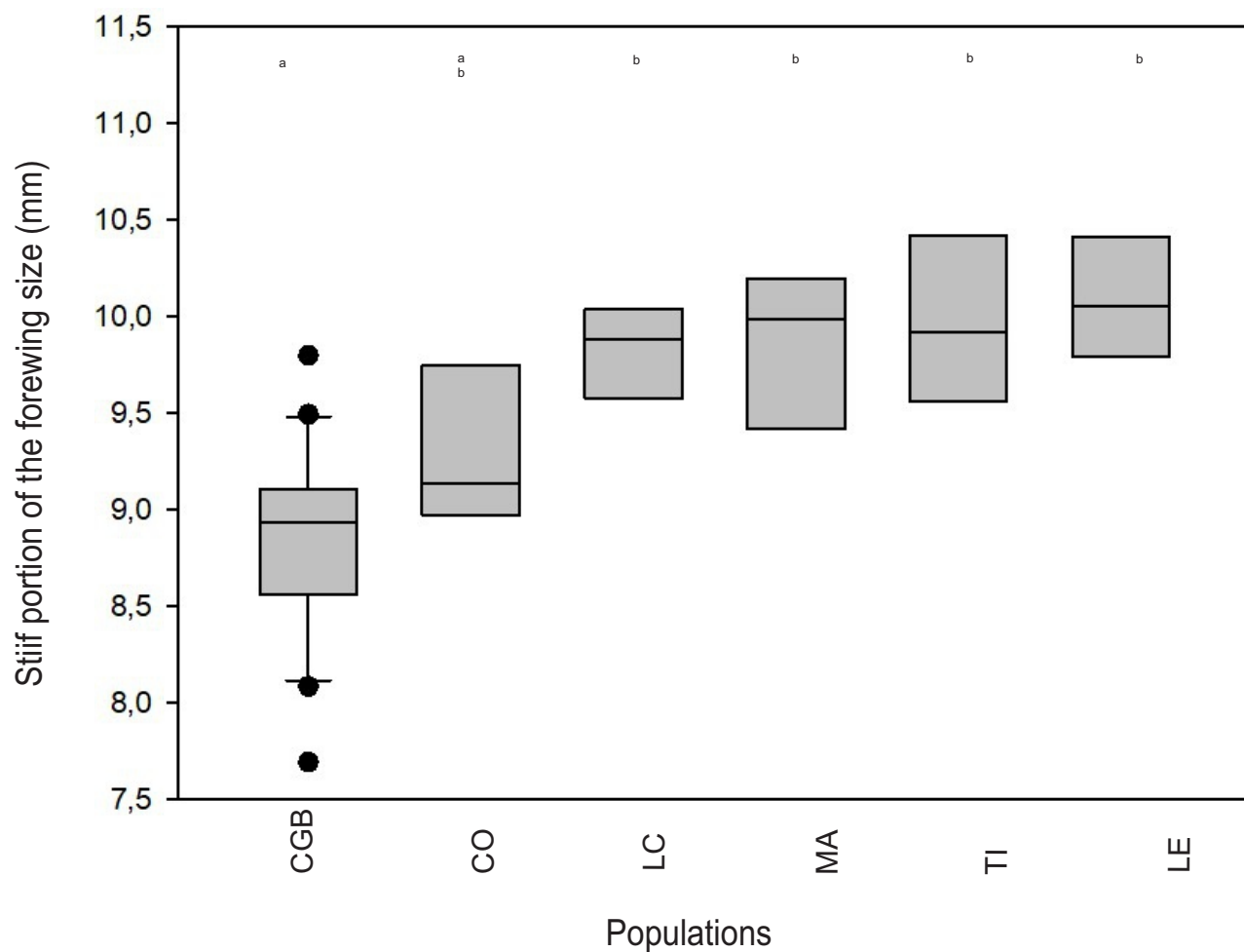

F

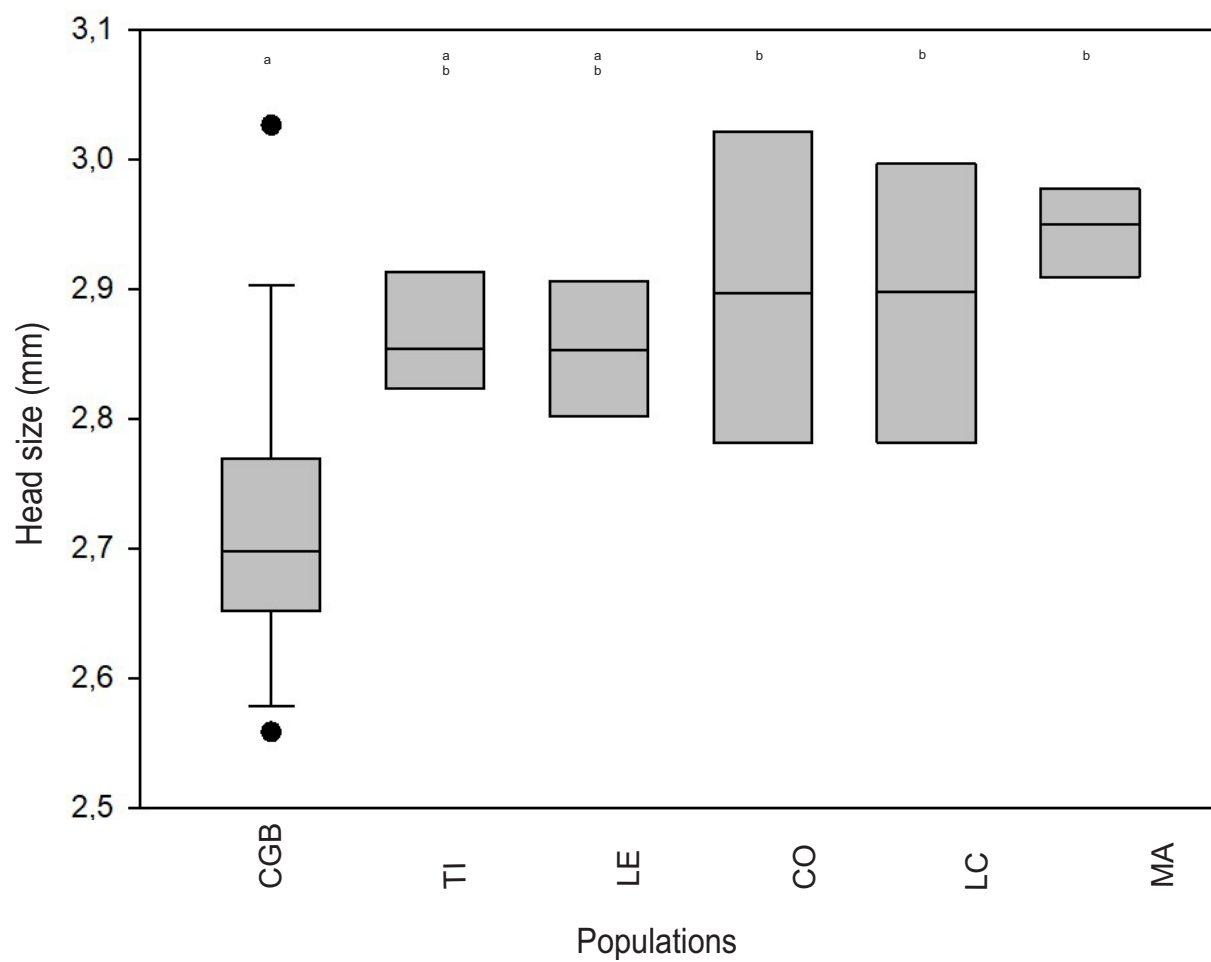

G

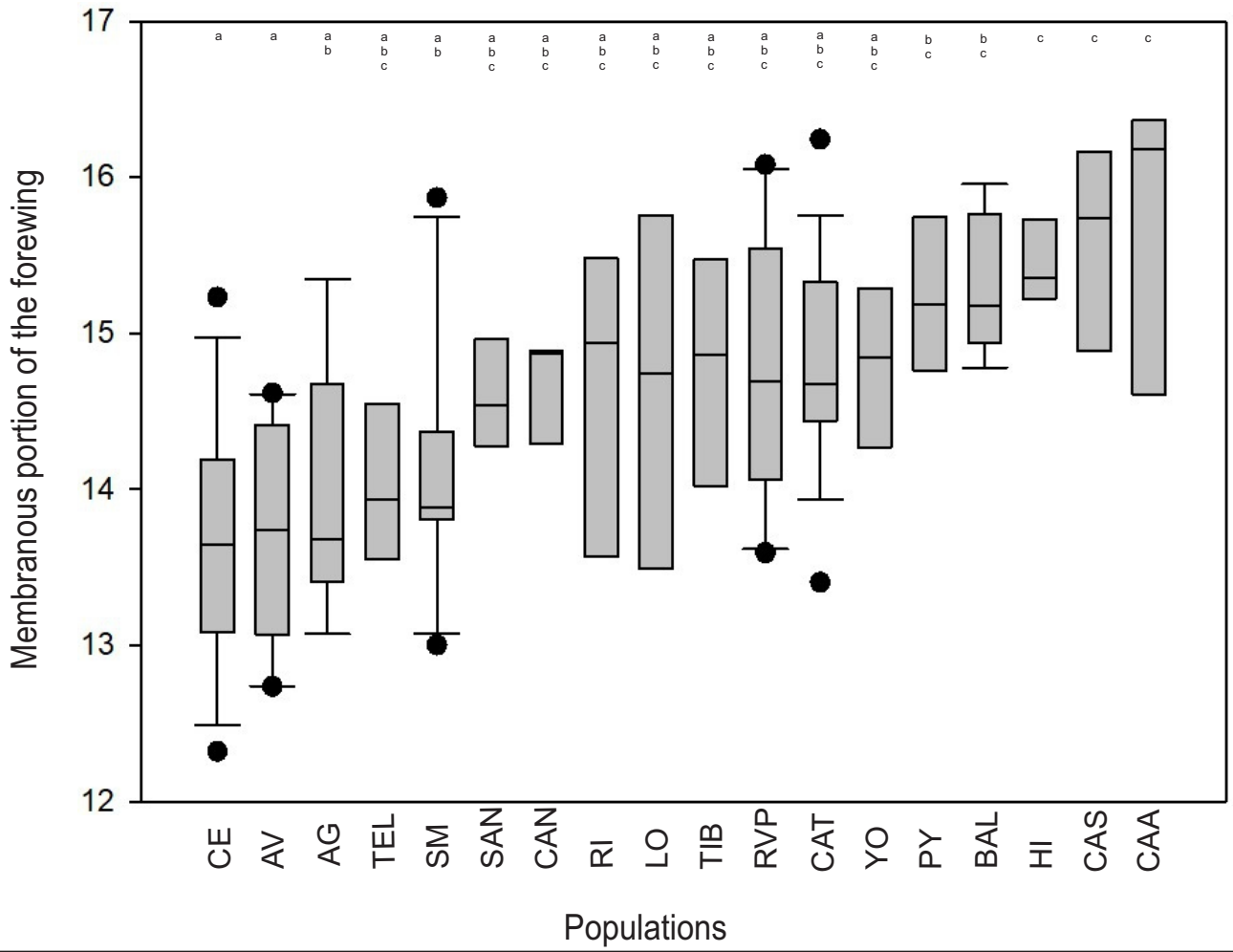

H

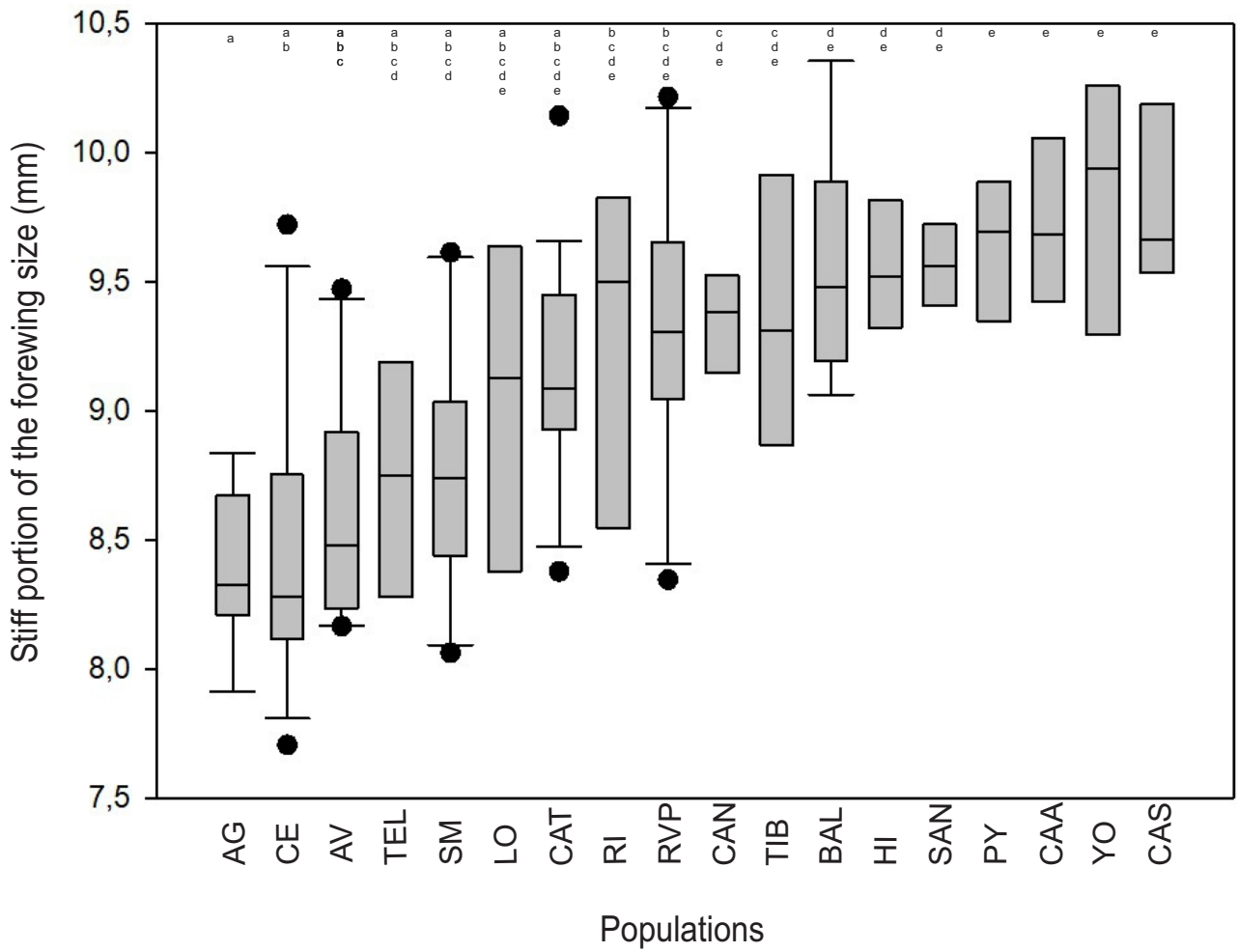

Supplement: Supplementary Material 1 — Box plot (median and standard deviation) of the size component (centroid size) of the membranous portion of the forewing (A), stiff portion of the forewing (B) across the species-level distribution range of Triatoma garciabesi. Forewing (C), membranous portion of forewing (D), stiff portion of forewing (E), head (F) of the Eastern lineage and membranous (G) and stiff portions of forewing (H) of the Western lineage are also shown. The solid horizontal line represents the median. Different letters above boxes indicate statistical difference at P < 0.05 (Tukey tests). Population codes as in Table 1 . [file DataSheet1.pdf]
