# Supplementary material for: Bug off or bug out: mapping flight secrets of Triatoma garciabesi (Hemiptera: Reduviidae) through climate, geography, and greenery
Source: Front Insect Sci. 2025 Jan 28;5:1532298. doi: 10.3389/finsc.2025.1532298 (PMC11810922; doi:10.3389/finsc.2025.1532298)
Supplement: Supplementary Material 2 — Matrices of pairwise Procrustes distances and pairwise permutation tests among all pairs of populations for the shape components of forewing, membranous and stiff portions of the forewing and head for the species-level distribution range of Triatoma garciabesi. Population codes as in Table 1 . [file DataSheet2.pdf]

A. Forewing shape for the Eastern lineage of T. garciabesi

Procrustes distances among populations:

1. TI					
2. CO	0.04				
3. CGB	0.0544	0.0555			
4. LE	0.0323	0.0429	0.0342		
5. LC	0.0241	0.0377	0.0458	0.0313	
6. MA	0.0344	0.0384	0.0393	0.0197	0.0307

P-values from permutation tests (10000 permutation rounds) for Procrustes distances among groups:

1. TI					
2. CO	0.0032				
3. CGB	<.0001	<.0001			
4. LE	0.0071	<.0001	0.0006		
5. LC	0.1323	0.0128	0.0007	0.0103	
6. MA	0.0029	0.0056	<.0001	0.2763	0.0255

B. Membranous portion of the forewing shape for the Eastern lineage of T. garciabesi

Procrustes distances among populations:

1. TI					
2. CO	0.0311				
3. CGB	0.0231	0.0383			
4. LE	0.023	0.0173	0.0241		
5. LC	0.0159	0.03	0.0222	0.0217	
6. MA	0.031	0.0227	0.0266	0.0135	0.0297

P-values from permutation tests (10000 permutation rounds) for Procrustes distances among groups:

1. TI					
2. CO	<.0001				
3. CGB	<.0001	<.0001			
4. LE	<.0001	<.0001	<.0001		
5. LC	<.0001	<.0001	<.0001	<.0001	
6. MA	<.0001	<.0001	<.0001	<.0001	<.0001

C. Stiff portion of the forewing shape for the Eastern lineage of T. garciabesi

1. TI					
2. CO	0.0651				
3. CGB	0.0342	0.0689			
4. LE	0.0273	0.047	0.0296		
5. LC	0.0336	0.0684	0.0276	0.0315	
6. MA	0.035	0.0529	0.0338	0.0217	0.0366

P-values from permutation tests (10000 permutation rounds) for Procrustes distances among groups:

1. TI					
2. CO	<.0001				
3. CGB	<.0001	<.0001			
4. LE	<.0001	<.0001	<.0001		
5. LC	<.0001	<.0001	<.0001	<.0001	
6. MA	<.0001	<.0001	<.0001	<.0001	<.0001

D. Head shape for the Eastern lineage of T. garciabesi

Procrustes distances among groups:

1. TI					
2. CO	0.0289				
3. CGB	0.0345	0.0344			
4. LE	0.0214	0.0283	0.0261		
5. LC	0.0216	0.0293	0.0232	0.0183	
6. MA	0.0173	0.0241	0.0259	0.019	0.0176

P-values from permutation tests (10000 permutation rounds) for Procrustes distances among groups:

1. TI			
2. CO	0.0788		
3. CGB	0.0005	0.0004	
4. LE	0.1559	0.009	0.0006

5. LC	0.2957	0.0341	0.0126	0.3129	
6. MA	0.6956	0.4471	0.0451	0.4609	0.7076
