# Supplementary material for: Bug off or bug out: mapping flight secrets of Triatoma garciabesi (Hemiptera: Reduviidae) through climate, geography, and greenery
Source: Front Insect Sci. 2025 Jan 28;5:1532298. doi: 10.3389/finsc.2025.1532298 (PMC11810922; doi:10.3389/finsc.2025.1532298)
Supplement: Supplementary Material 3 — Matrices of pairwise Procrustes distances and pairwise permutation tests among all pairs of populations for the shape components of forewing, membranous and stiff portions of the forewing and head for the Eastern lineage distribution range of Triatoma garciabesi. Numbers close to the nodes are 1,000 replicates of bootstrap values. [file DataSheet3.pdf]

A. Forewing size at the species distribution level of Triatoma garciabesi

r-PLS: 0.4163

Effect Size (Z): 4.84001

P-value: 1e-04

Correlation with climatic variables:

```
             [,1]
LAT   -0.383781624
LONG  -0.299726046
ALT   -0.021413935
NDVI  -0.058224193
LAILV -0.008558015
LAIHV -0.169607257
TM    -0.142774791
TMMMC  0.220573717
TMMMF -0.166213331
RH    -0.533993393
PREC  -0.338464437
VELOC  0.436571794
DV    -0.20789699
```

B. Forewing size for the Eastern lineage distribution range of Triatoma garciabesi

r-PLS: 0.7105

Effect Size (Z): 4.34527

P-value: 1e-04

Correlation with climatic variables:

```
            [,1]
LAT   -0.33005924
LONG  -0.15809836
ALT   -0.21913822
NDVI  -0.12381353
LAILV -0.29164352
LAIHV  0.24302300
TM    -0.30201611
TMMMC -0.06538794
TMMMF -0.39203237
RH     0.29892601
PREC  -0.18812421
VELOC -0.34495734
DV     0.40703390
```

C. Forewing size for the Western lineage distribution range of Triatoma garciabesi

r-PLS: 0.4295

Effect Size (Z): 4.28059

P-value: 1e-04

Correlation with climatic variables:

```
            [,1]
LAT   -0.48769412
LONG  -0.30088109
ALT   -0.11737659
NDVI  -0.07554850
LAILV -0.09527876
LAIHV -0.03293319
TM    -0.18631830
TMMMC  0.18352424
TMMMF -0.27318219
RH    -0.42746613
PREC  -0.37955838
VELOC  0.40872475
DV    -0.07145349
```

D. Membranous portion of the forewing size for the species-level distribution range of Triatoma garciabesi

r-PLS: 0.4142

Effect Size (Z): 4.75243

P-value: 1e-04

Correlation with climatic variables:

```
               [,1]
LAT   -0.3722598588
LONG  -0.2011538403
ALT   -0.0722169050
NDVI  -0.0007275207
LAILV  0.1474645969
LAIHV -0.2358943542
TM    -0.0821905660
TMMMC  0.2027826676
TMMMF -0.1767069220
RH    -0.5075812444
PREC  -0.3431026920
VELOC  0.4075275088
DV    -0.3432399781
```

E. Membranous portion of the forewing size for the Eastern lineage distribution range of Triatoma garciabesi

r-PLS: 0.7508

Effect Size (Z): 4.60458

P-value: 1e-04

Correlation with climatic variables:

```
               [,1]
LAT   -0.2958494258
LONG  -0.2219804224
ALT   -0.1674318775
NDVI  -0.0956494489
LAILV -0.3200006985
LAIHV  0.2668281709
TM    -0.2560666690
TMMMC  0.0007530453
TMMMF -0.4061324379
RH     0.2437342984
PREC  -0.2443118112
VELOC -0.3793680137
DV     0.3986086006
```

F. Membranous portion of the forewing size for the Western lineage distribution range of Triatoma garciabesi

r-PLS: 0.4677

Effect Size (Z): 4.75707

P-value: 1e-04

Correlation with climatic variables:

```
             [,1]
LAT   -0.45858021
LONG  -0.31087668
ALT   -0.08002721
NDVI  -0.09137608
LAILV -0.09246965
LAIHV -0.08229719
TM    -0.13461748
TMMMC  0.22075943
TMMMF -0.29050759
RH    -0.42244453
PREC  -0.46153181
VELOC  0.32204128
DV    -0.12864540
```

G. Stiff portion of the forewing size for the species-level distribution range of Triatoma garciabesi

r-PLS: 0.5168

Effect Size (Z): 6.30417

P-value: 1e-04

Correlation with climatic variables:

```
             [,1]
LAT   -0.40143413
LONG  -0.33917509
ALT    0.04731738
NDVI  -0.13865433
LAILV -0.02275080
LAIHV -0.19849459
TM    -0.17451043
TMMMC  0.21557288
TMMMF -0.24387519
RH    -0.44169292
PREC  -0.37933871
VELOC  0.38682258
DV    -0.19340730
```

H. Stiff portion of the forewing size for the Eastern lineage distribution range of Triatoma garciabesi

r-PLS: 0.7043

Effect Size (Z): 4.24784

P-value: 1e-04

Correlation with climatic variables:

```
             [,1]
LAT   -0.33602821
LONG  -0.15940710
ALT   -0.22940442
NDVI  -0.11801499
LAILV -0.30530566
LAIHV  0.24676683
TM    -0.27932188
TMMMC -0.06806526
TMMMF -0.40347310
RH     0.27834640
PREC  -0.17669135
VELOC -0.34750975
DV     0.40756762
```

I. Stiff portion of the forewing size for the Western lineage distribution range of Triatoma garciabesi

r-PLS: 0.5535

Effect Size (Z): 5.55297

P-value: 1e-04

Correlation with climatic variables:

```
             [,1]
LAT   -0.47692526
LONG  -0.32926390
ALT   -0.03942591
NDVI  -0.15110489
LAILV -0.04689282
LAIHV -0.10090308
TM    -0.20041888
TMMMC  0.17717861
TMMMF -0.32887261
RH    -0.36441477
PREC  -0.40391442
VELOC  0.36755678
DV    -0.12884085
```

J. Head size for the species-level distribution range of Triatoma garciabesi

r-PLS: 0.5163

Effect Size (Z): 5.92309

P-value: 1e-04

Correlation with climatic variables:

```
            [,1]
LAT   -0.46627871
LONG  -0.33656855
ALT    0.04477364
NDVI  -0.14788084
LAILV  0.09331294
LAIHV -0.14473547
TM    -0.31510103
TMMMC  0.08484524
TMMMF -0.35816779
RH    -0.30290339
PREC  -0.21382922
VELOC  0.39598630
DV    -0.29449381
```

L. Head size for the Eastern lineage distribution range of Triatoma garciabesi

r-PLS: 0.6378

Effect Size (Z): 3.70595

P-value: 1e-04

Correlation with climatic variables:

```
             [,1]
LAT   -0.36770575
LONG  -0.04610474
ALT   -0.27553156
NDVI  -0.19132201
LAILV -0.23921033
LAIHV  0.21804006
TM    -0.37490491
TMMMC -0.15880845
TMMMF -0.32110470
RH     0.36944606
PREC  -0.09652457
VELOC -0.26957789
DV     0.39751776
```

M. Head size for the Western lineage distribution range of Triatoma garciabesi

r-PLS: 0.5439

Effect Size (Z): 5.27721

P-value: 1e-04

Correlation with climatic variables:

```
             [,1]
LAT   -0.49024729
LONG  -0.38478079
ALT    0.01438022
NDVI  -0.21614076
LAILV  0.10130726
LAIHV -0.08578627
TM    -0.32934976
TMMMC  0.05286002
TMMMF -0.38395956
RH    -0.22873287
PREC  -0.21635670
VELOC  0.36326511
DV    -0.23932070
```
