# Supplementary material for: Bug off or bug out: mapping flight secrets of Triatoma garciabesi (Hemiptera: Reduviidae) through climate, geography, and greenery
Source: Front Insect Sci. 2025 Jan 28;5:1532298. doi: 10.3389/finsc.2025.1532298 (PMC11810922; doi:10.3389/finsc.2025.1532298)
Supplement: Supplementary Material 4 — Matrices of pairwise Procrustes distances and pairwise permutation tests among all pairs of populations for the shape components of forewing, membranous and stiff portions of the forewing and head for the Western lineage distribution range of Triatoma garciabesi. Numbers close to the nodes are 1,000 replicates of bootstrap values. [file DataSheet4.pdf]

A. Forewing shape for the species-level distribution range of Triatoma garciabesi

r-PLS: 0.5641

Effect Size (Z): 5.45325

P-value: 1e-04

Explained variance (%)

1: 79.31   2: 14.77

Correlation with climatic variables:

	[,1]	[,2]
LAT	-0.31713953	-0.25563601
LONG	-0.43818466	0.01460054
ALT	0.25891960	-0.06767681
NDVI	-0.37156427	0.25721443
LAILV	-0.38446443	0.52424250
LAIHV	-0.17204345	-0.26051614
TM	-0.23975476	-0.18499861
TMMMC	0.03552936	-0.11283499
TMMMF	-0.32470418	-0.24576991
RH	-0.20635608	-0.29465979
PREC	-0.34031367	0.03199505
VELOC	-0.00200415	0.21458896
DV	-0.04824703	-0.52890757

B. Forewing shape for the Eastern lineage distribution range of Triatoma garciabesi

r-PLS: 0.8586

Effect Size (Z): 5.40299

P-value: 1e-04

Explained variance (%)

1: 87.54   2: 10.07

Correlation with climatic variables:

	[,1]	[,2]
LAT	0.3518089	0.14825257
LONG	0.1047692	-0.43286169
ALT	0.2334562	0.26886669
NDVI	0.2771007	0.30775310
LAILV	0.3268397	-0.17371735
LAIHV	-0.2329782	0.21180290
TM	0.3563018	0.21922326
TMMMC	0.1130310	0.40088797
TMMMF	0.2696443	-0.25803136
RH	-0.2998176	-0.21819205
PREC	0.1567188	-0.37567177
VELOC	0.3125735	-0.28621637
DV	-0.3850658	0.05104344

C. Forewing shape for the Western lineage distribution range of Triatoma garciabesi

r-PLS: 0.5608

Effect Size (Z): 4.80815

P-value: 1e-04

Explained variance (%)

1: 83.36   2: 8.11

Correlation with climatic variables:

	[,1]	[,2]
LAT	-0.39490869	-0.02887200
LONG	-0.37272697	-0.07582868

```
ALT    0.17114203  0.09694460
NDVI  -0.32733625 -0.12962303
LAILV -0.13750648 -0.54415899
LAIHV -0.27298171  0.24188237
TM    -0.29078738 -0.12101886
TMMMC -0.10493887 -0.06484450
TMMMF -0.40607134  0.08158302
RH    -0.22771938 -0.42669631
PREC  -0.25328655  0.15422463
VELOC  0.05362026  0.17179631
DV    -0.30968910  0.59306394
```

D. Membranous portion of the forewing shape for the species-level distribution range of Triatoma garciabesi

r-PLS: 0.4524

Effect Size (Z): 4.42055

P-value: 1e-04

Explained variance (%)

1: 78.86   2: 12.42

Correlation with climatic variables:

```
             [,1]        [,2]
LAT   -0.35773756 -0.20812029
LONG  -0.44545504  0.10418567
ALT    0.24202527 -0.16500431
NDVI  -0.33597259  0.30450928
LAILV -0.25751803  0.58399257
LAIHV -0.22468499 -0.14306999
TM    -0.31666130 -0.13127648
TMMMC  0.05631122 -0.01141123
TMMMF -0.30128428 -0.19600396
RH    -0.32613751 -0.26106391
PREC  -0.25513916  0.08280510
VELOC  0.12715962  0.31256602
DV    -0.05909459 -0.48566736
```

E. Membranous portion of the forewing shape for the Eastern lineage distribution range of Triatoma garciabesi

-PLS: 0.6997

Effect Size (Z): 3.93118

P-value: 1e-04

#Varianza explicada (%)

1: 86.18   2: 12.55

#Relacion con variables climaticas

```
             [,1]        [,2]
LAT    0.39511548  0.01010391
LONG  -0.12044627  0.43954591
ALT    0.36890197 -0.20078201
NDVI   0.30031743  0.12606943
LAILV  0.18366050  0.38367161
LAIHV -0.11241949 -0.22250061
TM     0.38458275  0.03872498
TMMMC  0.30553051 -0.32666411
TMMMF  0.19523430  0.14600059
RH    -0.37676477  0.10426480
PREC  -0.07345607  0.46685128
VELOC  0.14018428  0.39810199
DV    -0.33220411 -0.19221931
```

F. Membranous portion of the forewing shape for the Western lineage distribution range of Triatoma garciabesi

r-PLS: 0.4641

Effect Size (Z): 3.69842

P-value: 2e-04

#Varianza explicada (%)

1: 84.07   2: 10.02

#Relacion con variables climaticas

```
             [,1]         [,2]
LAT   -0.42082003 -0.075792435
LONG  -0.42303062  0.035209048
ALT    0.16426865 -0.271988886
NDVI  -0.34888913 -0.003048975
LAILV -0.12023709 -0.282727801
LAIHV -0.25020442  0.237420146
TM    -0.35700989  0.100966114
TMMMC -0.03787961  0.413829094
TMMMF -0.35349346  0.091308702
RH    -0.28682121 -0.295885612
PREC  -0.17328869 -0.102197943
VELOC  0.17318866  0.559176404
DV    -0.14849354  0.426928945
```

G. Stiff portion of the forewing shape for the species-level distribution range of Triatoma garciabesi

r-PLS: 0.3917

Effect Size (Z): 3.68776

P-value: 1e-04

Explained variance (%)

1: 61.92   2: 23.57

Correlation with climatic variables:

```
             [,1]        [,2]
LAT    0.38557746  0.15313937
LONG   0.13069103  0.45846248
ALT    0.18963781 -0.29012627
NDVI  -0.07667148  0.38235118
LAILV -0.21304917  0.55989246
LAIHV -0.15565037  0.07973633
TM     0.10783101  0.11636790
TMMMC -0.37276509 -0.09361477
TMMMF  0.36496974  0.06343244
RH     0.14617873  0.13574267
PREC   0.36490575  0.33381002
VELOC -0.44222661  0.11447885
DV     0.29819259 -0.21648986
```

H. Stiff portion of the forewing shape for the Eastern lineage distribution range of Triatoma garciabesi

r-PLS: 0.7462

Effect Size (Z): 4.16614

P-value: 1e-04

Explained variance (%)

1: 77.01   2: 21.22

Correlation with climatic variables:

```
            [,1]         [,2]
LAT   -0.37779961 -0.104620390
LONG   0.20437058 -0.398713865
ALT   -0.37527343  0.075116961
NDVI  -0.36973260  0.004864704
LAILV -0.14061905 -0.376086276
LAIHV  0.04901499  0.350722165
TM    -0.38941364 -0.065341573
```

TMMMC	-0.35637037	0.238395575
TMMMF	-0.07843915	-0.312635217
RH	0.36386044	-0.001300169
PREC	0.14824810	-0.392327893
VELOC	-0.05653171	-0.420939742
DV	0.27097659	0.264599315

I. Stiff portion of the forewing shape for the Western lineage distribution range of Triatoma garciabesi

r-PLS: 0.4314

Effect Size (Z): 3.77169

P-value: 1e-04

Explained variance (%)

1: 78.64   2: 10.60

Correlation with climatic variables:

	[,1]	[,2]
LAT	0.41303431	0.23839744
LONG	0.14025924	0.30716746
ALT	0.20527472	-0.26971681
NDVI	0.01694002	0.17928965
LAILV	-0.29413291	0.08319285
LAIHV	-0.14595679	0.25312469
TM	0.13400813	0.31160378
TMMMC	-0.32781474	0.41708889
TMMMF	0.35774249	0.28993029
RH	0.05809327	0.11890181
PREC	0.36365930	0.02316621
VELOC	-0.43999323	0.37307489
DV	0.27725520	0.40490850

J. Head shape for the species-level distribution range of Triatoma garciabesi

r-PLS: 0.3986

Effect Size (Z): 3.39862

P-value: 2e-04

Explained variance (%)

1: 77.04 2: 12.90

Correlation with climatic variables:

	[,1]	[,2]
LAT	0.49723395	0.13181185
LONG	0.34699252	-0.26060171
ALT	-0.14492224	0.01036216
NDVI	0.32311582	-0.11355740
LAILV	0.19912495	-0.30968691
LAIHV	0.03465868	-0.36696111
TM	0.39724291	0.31156155
TMMMC	0.09194181	0.51866623
TMMMF	0.45653979	0.20051279
RH	0.12579041	-0.23007542
PREC	0.22272496	-0.43626744
VELOC	-0.07920386	0.12616903
DV	0.14039105	0.07529972

L. Head shape for the Eastern lineage distribution range of Triatoma garciabesi

r-PLS: 0.6079

Effect Size (Z): 2.78493

P-value: 0.0022

Explained variance (%)

1: 75.45   2: 20.98

Correlation with climatic variables:

	[,1]	[,2]
LAT	0.3595772	-0.17873194
LONG	0.1055637	0.42189852
ALT	0.2311967	-0.30689696
NDVI	0.2691114	-0.27519465
LAILV	0.3427884	0.17176055
LAIHV	-0.2344261	-0.27392929
TM	0.3445622	-0.18749612
TMMMC	0.1070697	-0.40559014
TMMMF	0.2722926	0.19653029
RH	-0.2818184	0.20800622
PREC	0.1441405	0.39184285
VELOC	0.3174190	0.28292548
DV	-0.3948987	-0.02152203

M. Head shape for the Western lineage distribution range of Triatoma garciabesi

r-PLS: 0.4081

Effect Size (Z): 2.64515

P-value: 0.0033

Explained variance (%)

1: 84.97   2: 6.32

Correlation with climatic variables:

	[,1]	[,2]
LAT	0.46221986	-0.05836601
LONG	0.39667652	0.10413063
ALT	-0.16001410	-0.39835128
NDVI	0.23873623	0.09626487
LAILV	0.07662046	-0.05060540
LAIHV	0.07077380	-0.26069734
TM	0.38823490	0.33627252
TMMMC	0.11193216	0.42905721
TMMMF	0.44656687	-0.07161113
RH	0.24127100	-0.05472532
PREC	0.18758841	-0.51304438
VELOC	-0.09508801	0.04576538
DV	0.26533275	-0.42032633
